# Development of a Therapeutic Video Game With the MDA Framework to Decrease Anxiety in Preschool-Aged Children With Acute Lymphoblastic Leukemia: Mixed Methods Approach

**DOI:** 10.2196/37079

**Published:** 2022-08-22

**Authors:** Dai-Jie Yang, Meng-Yao Lu, Chi-Wen Chen, Pei-Ching Liu, I-Ching Hou

**Affiliations:** 1 College of Nursing National Yang Ming Chiao Tung University Taipei City Taiwan; 2 Department of Nursing National Taiwan University Hospital Taipei City Taiwan; 3 Department of Pediatrics National Taiwan University Hospital Taipei City Taiwan; 4 Efficient Smart Care Research Center National Yang Ming Chiao Tung University Taipei Taiwan

**Keywords:** acute lymphoblastic leukemia, therapeutic video games, childhood cancer, preschoolers, anxiety

## Abstract

**Background:**

Preschool-aged children with acute lymphoblastic leukemia (ALL) receive long-term treatment according to the Taiwan Pediatric Oncology Group (TPOG)–ALL 2013 protocol. Severe anxiety and noncompliance ahead of frequent invasive therapies leads to an increase in health care costs. Previous studies have shown that therapeutic video games (TVGs) can decrease the anxiety experienced by children who are ill. To our knowledge, no existing TVG has been designed specifically for preschool-aged children with ALL in Taiwan.

**Objective:**

The purpose of this study was to develop a TVG using the popular Mechanics, Dynamics, and Aesthetics (MDA) framework for game design and to investigate the effect of this TVG on the reduction of therapy-related anxiety among preschool-aged children with ALL.

**Methods:**

This study used a mixed methods approach over three phases: (1) develop a TVG using the MDA framework, (2) test the reliability of the TVG among three certified children’s art therapists, and (3) evaluate the reduction of therapy-related anxiety among participants after using the TVG for 6 weeks, using a two-group, stratified randomized controlled trial at a medical center in northern Taiwan. Eligible preschool-aged children with ALL were randomly assigned 1:1 into an experimental group or a control group. The two groups of subjects received the same usual care, and only the experimental group had access to and used the TVG. The children’s anxiety responses were reported by their family caregivers using the face rating scale (FRS). Descriptive analyses, the Fisher exact test, the Pearson chi-square test, and the Mann-Whitney U test were used to statistically analyze the variables.

**Results:**

Six mechanics rules supported the dynamics of the TVG using four main features—character, nursery, tasks, and market—in order to complete all of the therapy-related anxiety reduction scenarios and to achieve eight aesthetics goals. The results of reliability test showed that participants found the TVG to be useful and trustworthy for preschool-aged children with ALL (Cronbach α=.98). A total of 15 participants were enrolled and randomly allocated to the experimental group (n=7) or the control group (n=8). The average number of TVG log-ins was 37.9 (SD 15.30, range 14-62) in the experimental group. The demographic data showed homogeneity across the two groups regarding age (3 to 5 years), sex (male), risk classification (standard risk), and treatment status (continuation therapy). The mean FRS score was 6.16 (SD 3.31) for the experimental group as compared to 7.45 (SD 2.71) for the control group (*P*=.04), which represented a significant difference between the groups at the 6-week follow-up.

**Conclusions:**

This research provides evidence that using a TVG can decrease anxiety in preschool-aged children with ALL in Taiwan. The TVG could be used to support clinical professionals before they perform invasive therapies. However, it is recommended to increase the statistical power for inference.

**Trial Registration:**

ClinicalTrials.gov NCT04199637; https://www.clinicaltrials.gov/ct2/show/NCT04199637

## Introduction

### Background

Acute lymphoblastic leukemia (ALL) is a type of cancer that constitutes a family of genetically heterogeneous lymphoid neoplasms derived from B- and T-lymphoid progenitors [1]; this type of cancer in humans shows an abnormal increase of leukocytes in the blood. ALL occurs mostly in children, particularly in those between 2 and 5 years of age, and its prevalence rate is about 30% to 40% in children worldwide [2]. In Taiwan, approximately 100 children aged 1 to 9 years are diagnosed with ALL every year, and more than half of them are preschoolers (ie, 3-5 years old) [3].

To obtain a definite diagnosis of ALL, bone marrow aspiration (BMA) must be performed. This is an invasive procedure performed by a physician to collect a small sample of bone marrow from a child’s hip bone, breastbone, or thigh bone under local anesthesia. Meanwhile, other body samples are also collected, such as peripheral blood cells, cerebrospinal fluid, and testicular cells. These results support the differential diagnosis of ALL, including the following: precursor B-cell ALL, early T-cell precursor (ETP) ALL, and precursor T-cell ALL with non-ETP [4].

The Taiwan Pediatric Oncology Group (TPOG), which consists of more than 60 pediatric hematologists and oncologists, classified children with ALL into one of three categories: standard risk, high risk, or very high risk. Classifications were based on the children’s presenting age, leukocyte count, presence of leukemia cells in the cerebrospinal fluid or testicular leukemia, immunophenotype, cytogenetics and molecular genetics, DNA index, and early response to therapy [2,4,5]. However, the risk categories will be reconfirmed according to the response to the protocol based on the minimal residual disease, which refers to a small number of cancer cells that remain in the body after treatment [6].

### The TPOG-ALL 2013 Protocol

The TPOG developed the first protocol in 1988 for childhood ALL; the protocol has since had several revisions [7]. At the beginning of our study, the TPOG-ALL 2013 protocol was used to treat children in Taiwan over four regimen stages: induction, consolidation, reinduction, and continuation. In this protocol, each stage consisted of different doses of antileukemic drugs (eg, prednisolone, vincristine, epirubicin, L-asparaginase, cyclophosphamide, cytarabine, and 6-mercaptopurine). The drugs were administrated by health professionals (eg, physicians and nurses) via intravenous (IV) injection, intramuscular (IM) injection, intrathecal (IT) injection, and oral intake. Multimedia Appendix 1 shows the summary of the protocol.

To facilitate the IV injection procedure, an artificial blood vessel, using a port-a-cath catheter system (PORT), would be implanted into the children’s left or right superior vena cava via surgery before the protocol. The needle with catheter—usually the Huber-point needles are used—would be inserted before IV injection and reinserted according to the clinical routine (eg, every 3 to 7 days) to prevent potential infection. The most common clinical signs and symptoms (ie, side effects) during the treatment were nausea, vomiting, mucositis, hair loss, bone marrow suppression, and pain [8]. Therefore, supportive medication and blood transfusions (BTs) will be provided as needed intravenously. In addition, to assess antileukemic response, a BMA will also be executed following the protocol, and the minimal residual disease will be monitored to guide the therapeutic choices [6].

### Therapy-Related Anxiety

A child’s ALL diagnosis can undoubtedly have a major impact on their family. Current intensive chemotherapy regimens have resulted in overall cure rates of 85% to 90% in children [1,7]. However, the discomfort caused by the side effects of the treatments [8] and the series of invasive therapies that must be endured during the disease journey all increase the anxiety of the children suffering from disease [9]. They also might resist professional treatment, leading to degeneration and overdependence behaviors [10].

In order to reduce therapy-related anxiety of children who are ill, many medical professionals have used cognitive and attentional distraction in order to control the anxiety of pediatric patients before, during, and after treatments [11,12]. However, medical staff have spent lots of time accompanying children who were ill, which was an invisible time cost, and the demand for clinical and medical labor has increased. Electronic technology products, such as computers, smartphones, and tablets, have become popular therapeutic adjuncts to traditional approaches (eg, drawing, role play, and toy operations). The games in portable electronic products have gradually become another aspect of children’s game activities. Many studies have applied video games or virtual reality to pediatric care [13-15]. However, this type of research is mostly limited to the use of general electronic games to divert attention, rather than specially designed therapeutic electronic games.

It has been clinically observed that the frequency of use of portable electronic products by preschool-aged children is extremely high. Almost every family caregiver will provide portable electronic products to children who are ill so they may watch videos. This shows that the development of related game products may be helpful for alleviating the anxiety experienced by children who are ill and may help them understand the purpose of treatment. Therefore, the purpose of this study was to develop a video game that can be played by preschool-aged children with ALL based on their cognitive development and game patterns, in order to understand how therapeutic video games (TVGs) can improve the anxiety response experienced by these children.

### The Mechanics, Dynamics, and Aesthetics Framework

The Mechanics, Dynamics, and Aesthetics (MDA) framework for game design and research was published by Hunicke et al in 2004 [16]. Mechanics describes the particular components of the game, at the level of data representation and algorithms (eg, rules). Dynamics describes the run-time behavior of the mechanics acting on the player inputs and each other’s outputs over time. Aesthetics describes the desirable emotional responses evoked in the player when the player interacts with the game system. These emotional responses include, but are not limited to, the taxonomy of sensation (eg, game as sense-pleasure), fantasy (eg, game as make-believe), narrative (eg, game as drama), challenge (eg, game as obstacle), fellowship (eg, game as social framework), discovery (eg, game as uncharted territory), expression (eg, game as self-discovery), and submission (eg, game as pastime). Each component of the MDA framework is causally linked by the game designers and provides the players with pleasure and fun perceptions [16]. When designing video games for preschool-aged children who are ill in order to decrease their therapy-related anxiety, the MDA framework could act as the guideline for the game designer.

## Methods

### Study Design

In this study, which began in 2018, our team conducted a mixed methods approach with three phases. In the first phase, the MDA framework [16] was adopted to design a TVG. In the second phase, a reliability test was applied before the TVG was used. In the third phase, a randomized controlled trial (RCT) was conducted to evaluate therapy-related anxiety experienced by preschool-aged children with ALL during their treatment according to the TPOG-ALL 2013 protocol. This study was registered at ClinicalTrials.gov (NCT04199637).

### Phase 1: Design of a TVG With the MDA Framework

To decrease therapy-related anxiety experienced by preschool-aged children with ALL, the research team cooperated with a Taiwan-based video game technology company to develop a TVG. Group meetings were held to reach a consensus regarding the MDA framework [16]. The game designer (DJY), who was also a registered nurse in the pediatric oncology ward, expressed that based on the knowledge of preschool-aged children’s cognitive-developmental stage (eg, preoperational), children 3 to 5 years of age show egocentrism, show anthropomorphism, think without logic, and lack conservation [17]. Thus, to facilitate aesthetics when playing the game, the TVG design should avoid mechanics and dynamics with complex logical (eg, calculation) or conservation (eg, comparison) aspects, but should provide an anthropomorphic role, easy and repeatable activities, and low-challenge tasks.

TVG development consensus meetings were held regularly (eg, once per week). The team first focused on the aesthetics expectations and dynamic features and then defined the mechanics data representations and algorithms. When the MDA framework was conceptually saturated and logically feasible, the game designer (DJY) provided a user interface draft for our TVG, including procedures for invasive therapies and required medical supplies (Multimedia Appendix 2), to the visual designer and programmer to help them design the game for preschool-aged children with ALL. Once the prototype and vital version of the TVG was produced, the research team tested and retested all the features, after which it was assessed during phase 2.

### Phase 2: TVG Reliability Test

To assess the reliability of the TVG, three certified children’s art therapists were invited to preview the TVG. After previewing the game, they completed a 12-item instrument containing two domains—usefulness (9 items) and trust (2 items)—with structured questions answered on a 5-point Likert scale ranging from 1 (strongly disagree) to 5 (strongly agree). A single open question allowed input of narrative feedback about the game. We performed the following to evaluate the usefulness and participants’ trust of the TVG: descriptive analysis of the 11 structured items, content analysis of the single narrative feedback item, and internal consistency (ie, Cronbach α) of the three therapists, in order to analyze reliability. The results were also used to optimize the TVG before phase 3.

### Phase 3: RCT to Measure the Effects of the TVG

#### Study Design and Setting

The TVG trial was a patient-blinded, parallel-group, stratified RCT with a 6-week follow-up evaluation. The study site was the medical center (eg, National Taiwan University Hospital) in northern Taiwan. Every patient at the study site received the same verbal comfort from nurses before and during the process of the invasive therapy. Some attention-shifting skills (eg, watching cartoons, listening to nursery rhymes and kids’ songs, and being given comforting toys) were also adopted, but these were nonstandardized and enabled by their caregivers and professionals to facilitate the therapy procedure. Therefore, the influence of the professionals’ care model on anxiety improvement was assumed to be similar for each patient in this study.

#### Ethical Considerations

This study followed the ethical principles of the Declaration of Helsinki [18]. This study was approved by the Institutional Review Board (IRB) of the National Taiwan University Hospital (IRB No. 201705014RINC) in Taiwan. Participation in the study was voluntary. Eligible participants were provided with one of two informed consent forms, according to their assigned groups. The consent forms included the same information about participant data that were collected regarding therapy-related anxiety responses. Only the consent forms for the experimental group provided briefing information about the TVG. Every participant was provided US $0.70 for each follow-up evaluation during the study.

#### Participant Recruitment, Sample Size, and Randomization

Preschool-aged children who met the following inclusion criteria were invited to participate in the study: (1) diagnosis of ALL and (2) aged 3 to 5 years. Exclusion criteria included the following: (1) has not undergone treatment according to the TPOG-ALL 2013 protocol, (2) did not have the PORT system inserted, (3) undergoing peripheral blood stem cell transplant treatment, (4) experiencing recurrent ALL, and (5) diagnosis of mental retardation, because of which TVG would not support their needs in this trial.

Participants were recruited by the researcher (DJY) and one trained research assistant in the pediatric hematology ward or outpatient clinic. Eligible patients were screened by the researcher (DJY) before recruitment in the study setting, according to the 2015-2016 Taiwan ALL incidence rate within each age and sex strata [3]. Next, within each age and sex strata, participants were randomly assigned 1:1 into one of the two study groups: control or experimental. For each strata, the randomization scheme was generated using an online randomization program [19].

#### Data Collection Procedures

Data collection by the study team took place from March 2018 to April 2020. Patients were blinded as to whether they were in the experimental or control group. Demographics data (eg, age, sex, diagnosis, risk classifications, and treatment stage) of the eligible participants were collected through the electronic health record at the study site. The TVG was only installed on the smartphones of the experimental group participants’ primary caregivers; they were then taught how to use it. Each participant in the experimental group could play the TVG at any time as needed (eg, no prompts or reminders from the study team were needed). To ensure that the patients in the control group were not receiving the intervention, the TVG web address and log-in account details were only provided by the research team who were, themselves, not able to access the game. Meanwhile, the experimental group caregivers were told, and agreed to, not to share the TVG log-in account details with any other children with ALL during the trial.

The 6-week follow-up evaluation data collection was completed via a web-based instrument following the invasive therapy. To prevent missing records, communication software (eg, Line) was used to remind the participant caregivers every week. All of the intervention group participants received the same TVG (eg, frozen version), and there were no technical problems associated with the TVG during the trial.

#### Therapy-Related Anxiety Response Report

In this study, therapy-related anxiety responses by the children were reported by their family caregivers through a web form that was installed on their mobile phones at enrollment. This form consisted of four items: invasive therapy date, invasive therapies received, face rating scale (FRS) score, and other responses. The second item—invasive therapies conducted—included six activities: buttocks injection, chemical medication administration, IT injection, BMA, PORT puncture, and blood transfusion. The third item–the FRS–included 10 faces with corresponding numbers: a smiling face with mouth open (ie, number 1) indicates not crying at all and a red face with two tears (ie, number 10) indicates severe crying. The fourth item—other responses—allowed family caregivers to note any observations about the participants in a free-text field. When the participant completed any one of six invasive therapies, the family caregiver would help them point out on the FRS the face that expressed their real feelings during that therapy. The average time that the caregivers spent completing the report was less than 5 minutes without any assistance.

#### Data Analysis

All reported data were coded and analyzed using SPSS (version 24; IBM Corp). Frequency and percentage as well as mean and SD were used as descriptive statistics for the demographic data and for FRS results. We compared the age, sex, risk classifications, treatment status, invasive therapies received, and FRS scores of participants in the control and experimental groups; the Fisher exact test and Pearson chi-square tests were used for categorical variables, and the Mann-Whitney *U* test was used for continuous variables. Significance was defined as *P*<.05. For the other responses, each report was categorized as positive or negative, which was defined by the research team with consensus. Positive responses were defined as the participants not crying or being just a little nervous at the beginning of the therapy. Negative responses were defined as the participants crying, reacting physically (eg, vomiting), or showing any resistance behaviors (eg, trying to run away).

## Results

### The Content of the TVG

**Figure 1:**
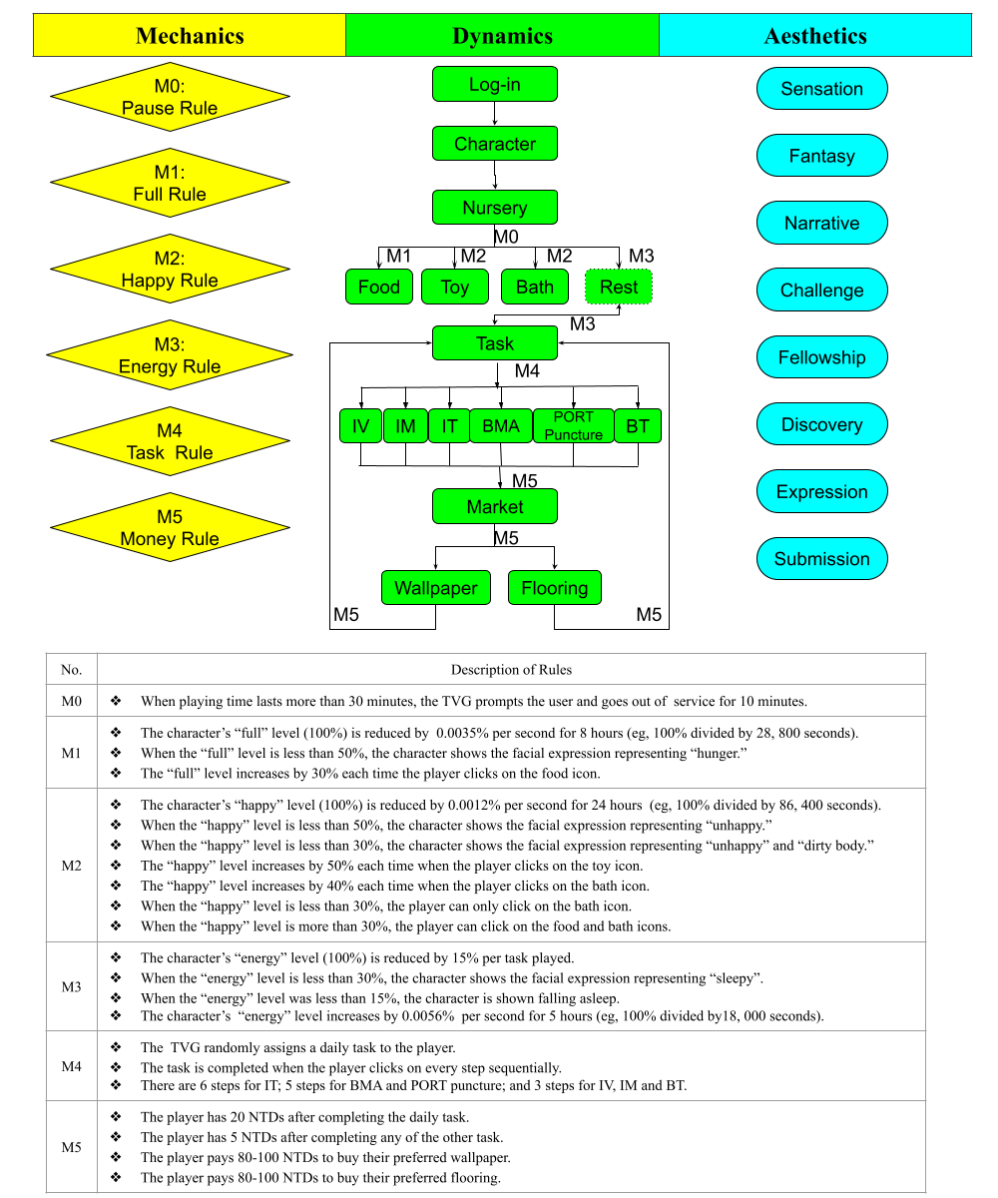
The therapeutic video game (TVG) with the MDA framework. BMA: bone marrow aspiration; BT: blood transfusion; IM: intramuscular; IT: intrathecal; IV: intravenous; MDA: Mechanics, Dynamics, and Aesthetics; NTD: New Taiwan Dollar; PORT: port-a-cath catheter system.

Following the MDA framework guideline (Figure 1), the TVG—named Saving the Animals’ Planet—was developed using PHP and MySQL and could be played on the website.

**Figure 2:**
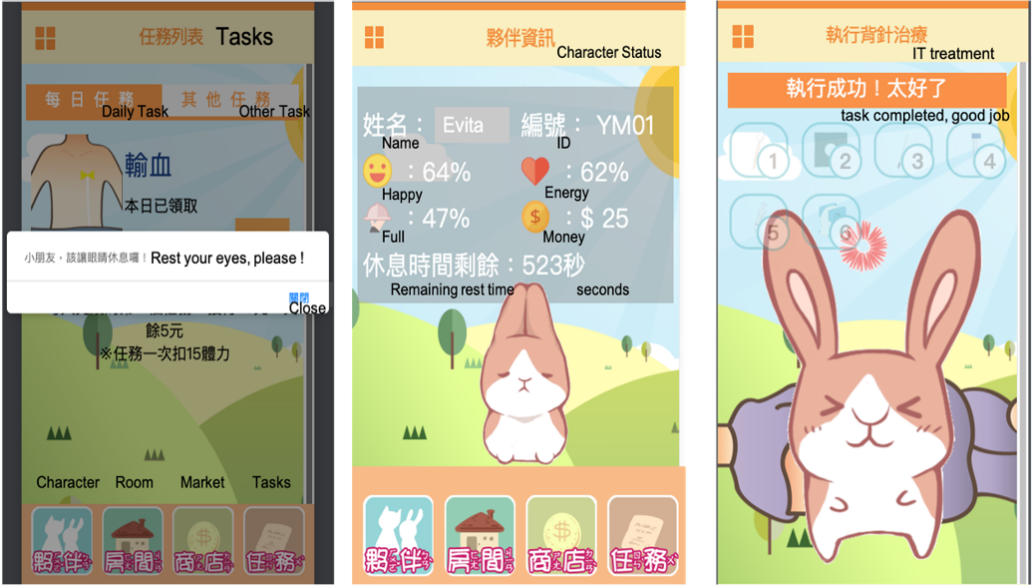
Screenshots showing the mechanics of the therapeutic video game. "Pause" rule (left); "happy," "full," "energetic," and "money" rules (middle); and "task" (ie, complete) rule (right).

With regard to the mechanics components, there were a total of six rules that included one or several data representations and algorithms to support the dynamics of the TVG. The “pause” rule was designed to remind the players to take breaks as needed. The “full,” “happy,” and “energetic” rules were designed to encourage the players to maintain good habits in their real life by eating food, playing with toys, and resting after playing the game. The “task” rule was designed to guide the players in familiarizing themselves with the procedures of the invasive therapies they would receive. The TVG made use of an encouraging voice (eg, “Good job!”) and images (eg, a character setting off fireworks) as positive feedback when the player completed any task. The “money” rule was designed to encourage the players to complete more tasks. Figure 2 shows some screenshots showing the mechanics of the game.

**Figure 3:**
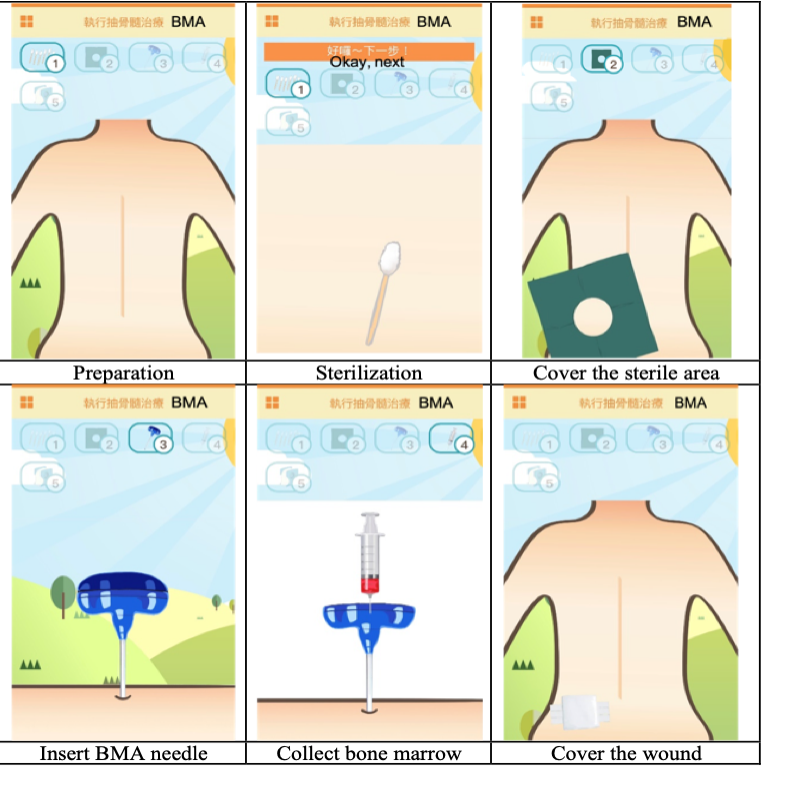
Screenshots showing the dynamics of the bone marrow aspiration (BMA) task.

With regard to dynamics, a total of four cartoon characters—rabbit, cat, bear, and dog—were designed, and the player could choose one character as their partner. There were four statuses for each character—full, happy, energetic, and money—to show the outcome resulting from how the player nursed their partner. The statuses were dynamically changed according to the rules of the mechanics. When the character was full of energy, it could perform any of six tasks: IV injection, IM injection, IT injection, BMA, BT, or PORT puncture. Using the BMA task as an example (Figure 3), the player would have the character follow the task rule by preparing and sterilizing the needle insertion area, then covering the sterile area with hole drape; inserting the BMA needle; collecting bone marrow; and covering the wound with gauze. After completing the task, the character would be rewarded with virtual coins, with which they could buy wallpaper or flooring from the TVG market to decorate their room.

With regard to aesthetics, a total of eight emotional responses were perceived after playing the TVG: sensation, fellowship, discovery, expression, challenge, narrative, submission, and fantasy (Table 1). The sensation response was the one that provided the most aesthetics features through the anthropomorphic characters, a pleasing melody, a tender voice, and warm color through the TVG interface. The nursery feature was based on the “full,” “happy,” and “energetic” rules to encourage the players to maintain good habits in their real life. The task feature elicited the most emotional responses, as a result of the role rehearsals that took place in the medical scenarios.

**Table 1:** The aesthetics of the therapeutic video game.

Feature	Aesthetics
Character	Sensation and fellowship
Nursery	Fellowship, sensation, discovery, and expression
Task	Discovery, challenge, narrative, sensation, expression, and fellowship
Market	Sensation, submission, discovery, expression, and fantasy

### The Reliability of the TVG

The three children’s art therapists were female, had master’s degrees, and were 27 to 39 years of age. Regarding the TVG, the mean usefulness score was 4.73 (SD 0.45) and the mean trust score was 4.66 (SD 0.51), which indicated that the users trusted the TVG and found it to be useful. The internal consistency (ie, Cronbach α) was .98, which indicated adequate reliability among the three therapists [20]. The narrative feedback showed that the TVG was able to (1) provide cute game elements and life simulations when completing the invasive therapy tasks, (2) have patients’ characters show them encouragement when completing the tasks, and (3) provide health education knowledge to patients’ family members.

### Randomization and Attrition

**Figure 4:**
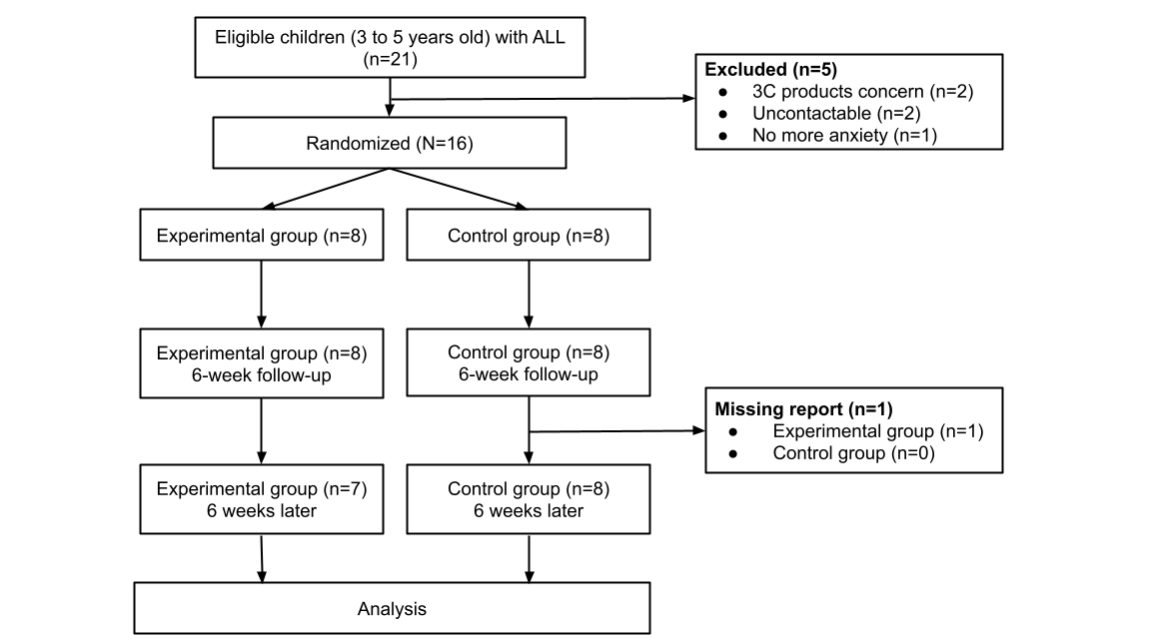
Randomized controlled trial study flowchart. 3C: computer, communication, and consumer electronics; ALL: acute lymphoblastic leukemia.

Randomization and attrition data were organized according to the CONSORT (Consolidated Standards of Reporting Trials) guidelines (Figure 4) [21]. Out of 21 eligible preschool-aged children with ALL, 2 family caregivers refused to participate in the trial because of concerns regarding their child using electronic products. Another 2 participants lost contact during the trial (eg, missed their clinic visiting time). An additional family caregiver said her child, who was 4 years and 10 months old, was no longer experiencing anxiety regarding receiving the invasive therapy. The remaining 16 eligible participants were enrolled and randomly allocated to the experimental group (n=8) or the control group (n=8). The recruitment rate was 76% (16/21). However, 1 participant from the experimental group dropped out because the report regarding their anxiety response was not submitted during the 6-week follow-up evaluation. A total of 7 participants remained in the experimental group, and 8 remained in the control group at the 6-week follow-up.

### Participant Demographics

The demographics from the two groups were similar (Table 2). Most children were 5 years old (7/15, 47%), most were male (10/15, 67%), most were classified as having standard risk (11/15, 73%), and the treatment status for most of them was at the continuation stage (10/15, 67%). There were no statistically significant differences between the groups, which shows homogeneity among participants in the experimental and control groups regarding demographic characteristics.

**Table 2:** Demographic characteristics of the participants.

Characteristic		Experimental group (n=7), n (%)	Control group (n=8), n (%)	Total participants (N=15), n (%)	*P* value	
**Age in years**						.95^a^
	3	3 (43)	2 (25)	5 (33)		
	4	0 (0)	3 (38)	3 (20)		
	5	4 (57)	3 (38)	7 (47)		
**Sex**						>.99^b^
	Male	5 (71)	5 (63)	10 (67)		
	Female	2 (29)	3 (38)	5 (33)		
**Risk classification**						>.99^b^
	Standard risk	5 (71)	6 (75)	11 (73)		
	High risk	2 (29)	2 (25)	4 (27)		
**Treatment status**						.94^c^
	Induction	2 (29)	1 (13)	3 (20)		
	Consolidation	0 (0)	1 (13)	1 (7)		
	Reinduction	0 (0)	1 (13)	1 (7)		
	Continuation	5 (71)	5 (63)	10 (67)		

^a^This *P* value was based on the Mann-Whitney *U* test.

^b^This *P* value was based on the Fisher exact test.

^c^This *P* value was based on the chi-square test (Kendall τb).

### Caregiver-Reported Invasive Therapies

According to the participants’ FRS reports that were recorded by their caregivers when they received invasive therapies (Table 3), the most frequent therapy was IM injection (experimental group: 25/67, 37%; control group: 27/69, 39%; *P*=.81), and the least frequent was BT (experimental group: 2/67, 3%; control group: 0/69, 0%; *P*=.29). The total number of times that invasive therapies were received was 67 for the experimental group and 69 for the control group. The Mann-Whitney *U* test showed no statistical difference between the two groups (*P*>.05 for all).

**Table 3:** Caregiver-reported invasive therapies.

Invasive therapy administered	Experimental group (n=7)				Control group (n=8)				*P* value^a^
	Times administered, n (%)	Range	Mean (SD)	Times administered, n (%)		Range	Mean (SD)		
IM^b^ injection (buttocks injection)	25 (37)	1-5	3.5 (1.6)	27 (39)		0-6	3.9 (1.3)	.81	
PORT^c^ puncture	17 (25)	0-6	2.8 (1.9)	18 (26)		1-4	2.3 (1.0)	.90	
IV^d^ injection	13 (19)	1-6	1.9 (1.9)	13 (19)		0-3	2.1 (0.4)	.50	
IT^e^ injection	6 (9)	0-2	2 (0)	8 (12)		0-2	1.1 (0.4)	.66	
BMA^f^	4 (6)	0-2	2 (0)	3 (4)		0-2	1.5 (0.7)	.77	
BT^g^	2 (3)	0-2	2 (0)	0 (0)		0-0	0 (0)	.29	
Total	67 (100)	6-15	9.6 (3.5)	69 (100)		2-11	8.6 (2.9)	>.99	

^a^This *P* value was based on the Mann-Whitney *U* test.

^b^IM: intramuscular.

^c^PORT: port-a-cath catheter system.

^d^IV: intravenous.

^e^IT: intrathecal.

^f^BMA: bone marrow aspiration.

^g^BT: blood transfusion.

### Actual TVG Usage in the Experimental Group

During the 6-week follow-up evaluation in the experimental group (n=7), the average number of log-ins was 37.9 (SD 15.30, range 14-62). The average number of invasive therapy tasks completed was 58.2 (SD 59.4, range 9-179). IT injection was the most-completed task (82/408, 20.1%; Table 4). The numbers of TVG log-ins and completed tasks were not significantly correlated to treatment status (ρ=–0.138, *P*=.70).

**Table 4:** Actual TVG usage in the experimental group (n=7).

Item		Log-ins or completed tasks				
		n (%)	Range	Mean (SD)		
TVG^a^ log-ins (n=265)		265 (100)	14-62	37.9 (15.30)		
**TVG tasks completed (n=408)**						
	Total	408 (100)	9-179	58.2 (59.4)		
	IM^b^ injection (buttocks injection)	79 (19.4)	1-28	11.3 (10.5)		
	PORT^c^ puncture	51 (12.5)	0-26	8.5 (9.3)		
	IV^d^ injection	66 (16.2)	0-33	11 (11.8)		
	IT^e^ injection	82 (20.1)	0-31	13.7 (11.7)		
	BMA^f^	63 (15.4)	0-28	10.5 (8.9)		
	BT^g^	67 (16.4)	0-33	11.1 (10.9)		

^a^TVG: therapeutic video game.

^b^IM: intramuscular.

^c^PORT: port-a-cath catheter system.

^d^IV: intravenous.

^e^IT: intrathecal.

^f^BMA: bone marrow aspiration.

^g^BT: blood transfusion.

### Primary Outcome: Children’s Anxiety Response Report

According to the participants’ FRS reports, the internal consistency (ie, Cronbach α) of FRS scores was .52, which indicated acceptable reliability [20]. Table 5 shows that the mean FRS score was 6.16 (SD 3.08) among 67 records in the experimental group, and the mean FRS score was 7.45 (SD 2.71) among 69 records in the control group. The Mann-Whitney *U* test showed a statistically significant difference (*P*=.04) between the two groups (Table 5). When receiving chemical medication therapy via IV injection, the FRS score in the experimental group (mean 3.62, SD 2.63) was significantly lower than in the control group (mean 5.85, SD 3.08; *P*=.04). The other five invasive therapies showed no difference between groups. The results regarding other responses showed that the experimental group had slightly more positive responses and fewer negative responses than the control group (Table 5).

**Table 5:** Children’s anxiety responses according to the FRS reports.

Item		Experimental group (n=7)			Control group (n=8)				*P* value		
		Records (n=67), n (%)	FRS^a^ score, range	FRS score, mean (SD)	Records (n=69), n (%)	FRS score, range	FRS score, mean (SD)				
**Invasive therapy**											
	Total	67 (100)	1-10	6.16 (3.31)	69 (100)	1-10	7.45 (2.71)	.04			
	IM^b^ injection (buttocks injection)	25 (37)	1-10	6.44 (2.97)	27 (39)	2-10	7.22 (2.83)	.42			
	PORT^c^ puncture	17 (25)	1-10	6.82 (3.82)	18 (26)	4-10	8.22 (2.16)	.63			
	IV^d^ injection	13 (19)	1-10	3.62 (2.63)	13 (19)	1-10	5.85 (3.08)	.04			
	IT^e^ injection	6 (9)	4-10	7.67 (2.58)	8 (12)	5-10	8.25 (2.12)	.64			
	BMA^f^	4 (6)	2-10	6.5 (3.69)	3 (4)	9-10	9.67 (0.58)	.20			
	BT^g^	2 (3)	7-10	8.50 (2.12)	0 (0)	N/A^h^	N/A	N/A			
**Other responses** ^i^											
	Positive	5 (7)	—	—	4 (6)	—	—	—			
	Negative	4 (6)	—	—	13 (19)	—	—	—			

^a^FRS: face rating scale.

^b^IM: intramuscular.

^c^PORT: port-a-cath catheter system.

^d^IV: intravenous.

^e^IT: intrathecal.

^f^BMA: bone marrow aspiration.

^g^BT: blood transfusion.

^h^N/A: not applicable; there were no control group records for BT therapy, so there were no FRS records for that group, and the *P* value could not be calculated.

^i^The other responses items were not evaluated using the FRS, so no values appear in the related columns.

## Discussion

### Principal Findings

Our team developed a TVG, which was the first game specifically designed for preschool-aged children with ALL in Taiwan in order to reduce their therapy-related anxiety during their long journey of cancer treatment (Multimedia Appendix 1). Using the MDA framework, each feature of the TVG—character, nursery, tasks, and market—was designed based on the characteristics of preschool-aged children (ie, egocentrism, anthropomorphism, thinking without logic, and lacking conservation) [17]. Next, six mechanics rules (eg, pause, happy, full, energetic, money, and task) supported the dynamics of the characters in the TVG in order to complete all of the therapy-related anxiety reduction scenarios and to achieve eight aesthetics goals: sensation, fellowship, discovery, expression, narrative, submission, challenge, and fantasy (Figure 1).

According to reliability tests among the three certified children’s art therapists, the results showed high agreement regarding the usefulness of the TVG (mean score 4.73, SD 0.45) and participants’ trust in the TVG (mean score 4.66, SD 0.51). Two items of feedback regarding the tasks—providing cute game elements and life simulations as well as encouragement provided by characters—were adopted within the MDA framework before the clinical validation trial. The recommendation to provide health education knowledge to patients’ family members was an important finding; in the future, we should think about how to involve other family members by providing additional benefits, such as this one, to those who adopt the TVG.

During the 6-week follow-up evaluation, the average number of log-ins (mean 37.9, SD 15.30) and completed tasks (mean 58.2, SD 59.4) in the experimental group showed participants’ acceptance and reliability in using TVG; these were not related to treatment status (ρ=–0.138, *P*=.70). The results indicated that the participants’ caregivers (eg, parents) trusted the TVG and were willing to allow their children to use it. Meanwhile, the TVG followed the TPOG-ALL 2013 protocol and was able to facilitate all stages of treatment.

Regarding the caregiver-reported children’s anxiety scale, the results showed that participants in the experimental group had a significant improvement in their FRS score (mean 6.16, SD 3.31 vs 7.45, SD 2.71; *P*=.04) and fewer negative responses (mean 4, SD 6 vs mean 13, SD 19) compared to the control group. This indicated that our TVG was able to reduce therapy-related anxiety.

### Comparison With Prior Work

According to a previous systematic review, improvement in therapy-related anxiety (Table 5) in our experimental group was consistent with the results of play therapy in reducing anxiety during hospital stays in different countries [22]. It was also similar to a study that found a reduction in preoperative anxiety among children aged 5 to 11 years in Jordan [23]. This may be because the TVG was designed for the preoperational stage of cognitive development of preschool-aged children, according to Piaget [17], which indicated that the participants could use the TVG easily without logical thinking. The content of the TVG also provided simulations of therapies based on real invasive therapies during ALL treatments, and it increased participants’ familiarity with these therapies by allowing them to play the TVG repeatedly.

Although the TVG was able to decrease therapy-related anxiety in children with ALL, there were still some negative concerns on the part of the family caregivers. A total of 3 family caregivers from the group of eligible participants were excluded from this trial; two-thirds of these caregivers (2/3, 67%) prevented their children from using video games. To our knowledge, there is an increasing amount of literature focused on pathological and nonpathological correlates of video game playing, with specific attention directed toward internet gaming disorder [24]. It may be the cause of the lack of trust in the TVG on the part of these family caregivers.

### Limitations

During the study protocol drafting phase, beginning in 2018, we adopted stratified random sampling and proposed a sample size of 34 in each group, according to the 2015-2016 Taiwan ALL incidence rate within each age and sex strata [3]. However, in Taiwan, the total number of births (ie, 165,000) was lower than the total number of deaths (ie, 173,000) for the first time in 2020 [25]. Therefore, this may have resulted in a lower ALL incidence rate among preschool-aged children during this trial (2018-2020). The statistical power for a sample size of 15 participants was 0.23, which is not adequate according to Cohen [26] and may affect the credibility of this study’s results.

Another limitation is related to the study’s participant blinding procedure. The primary caregivers of the participants knew their assigned group as a result of our informed consent process. To our knowledge, the effects of not blinding the patients’ caregivers, which may have caused self-report bias in the RCT study, were not clear. Therefore, the improvement in therapy-related anxiety after using our TVG was conservatively inferred.

### Conclusions

The purpose of this study was to investigate therapy-related anxiety responses by preschool-aged children with ALL in Taiwan after using an innovative TVG designed by our team. According to the results, the anxiety of the TVG users decreased after receiving cancer invasive therapies as compared to nonusers of the TVG. This may be because the TVG allows users to experience simulations of these invasive procedures through character role rehearsals. When the subjects received positive feedback after completing these tasks, they may have experienced cognitive and attentional distraction from their negative perceptions (ie, therapy-related anxiety).

### Implications

The results from this study led to the recommendation to include more cancer-related education in the TVG, such as medication taking, hand hygiene, and toothbrushing, to help preschool-aged children with ALL take better care of themselves. In addition, the TVG could also be applied to the other common cancers (eg, brain tumors or osteosarcomas) in children to help lessen their anxiety during difficult treatments. Health education knowledge could help family members take care of their children who are ill; this could be incorporated into the design of the next TVG.

## Appendix

Multimedia Appendix 1 TPOG-ALL 2013 protocol. ALL: acute lymphoblastic leukemia; TPOG: Taiwan Pediatric Oncology Group.

Multimedia Appendix 2 User interface draft for our therapeutic video game.

Multimedia Appendix 3 CONSORT-eHEALTH checklist (V 1.6.1).

## Abbreviations

ALL: acute lymphoblastic leukemia
BMA: bone marrow aspiration
BT: blood transfusion
CONSORT: Consolidated Standards of Reporting Trials
ETP: early T-cell precursor
FRS: face rating scale
IM: intramuscular
IRB: Institutional Review Board
IT: intrathecal
IV: intravenous
MDA: Mechanics, Dynamics, and Aesthetics
PORT: port-a-cath catheter system
RCT: randomized controlled trial
TPOG: Taiwan Pediatric Oncology Group
TVG: therapeutic video game
