# Correction: Development of a Therapeutic Video Game With the MDA Framework to Decrease Anxiety in Preschool-Aged Children With Acute Lymphoblastic Leukemia: Mixed Methods Approach

**DOI:** 10.2196/43211

**Published:** 2022-10-05

**Authors:** Dai-Jie Yang, Meng-Yao Lu, Chi-Wen Chen, Pei-Ching Liu, I-Ching Hou

**Affiliations:** 1 College of Nursing National Yang Ming Chiao Tung University Taipei City Taiwan; 2 Department of Nursing National Taiwan University Hospital Taipei City Taiwan; 3 Department of Pediatrics National Taiwan University Hospital Taipei City Taiwan; 4 Efficient Smart Care Research Center National Yang Ming Chiao Tung University Taipei Taiwan

In “Development of a Therapeutic Video Game With the MDA Framework to Decrease Anxiety in Preschool-Aged Children With Acute Lymphoblastic Leukemia: Mixed Methods Approach” (JMIR Serious Games 2022;10(3):e37079) the authors noted a few errors in Table 3.

In the originally published paper, the headings of subcolumns appeared as “FRS score, range” and “FRS score, mean (SD).” These headings have been corrected to “Range” and “Mean (SD),” respectively. The sequence of footnotes was revised accordingly. The updated version of Table 3 can be viewed below. The originally published Table 3 is in Multimedia Appendix 1.

**Table 3:** Caregiver-reported invasive therapies.

Invasive therapy administered	Experimental group (n=7)				Control group (n=8)				*P* value^a^
	Times administered, n (%)	Range	Mean (SD)	Times administered, n (%)		Range	Mean (SD)		
IM^b^ injection (buttocks injection)	25 (37)	1-5	3.5 (1.6)	27 (39)		0-6	3.9 (1.3)	.81	
PORT^c^ puncture	17 (25)	0-6	2.8 (1.9)	18 (26)		1-4	2.3 (1.0)	.90	
IV^d^ injection	13 (19)	1-6	1.9 (1.9)	13 (19)		0-3	2.1 (0.4)	.50	
IT^e^ injection	6 (9)	0-2	2 (0)	8 (12)		0-2	1.1 (0.4)	.66	
BMA^f^	4 (6)	0-2	2 (0)	3 (4)		0-2	1.5 (0.7)	.77	
BT^g^	2 (3)	0-2	2 (0)	0 (0)		0-0	0 (0)	.29	
Total	67 (100)	6-15	9.6 (3.5)	69 (100)		2-11	8.6 (2.9)	>.99	

^a^This *P* value was based on the Mann-Whitney *U* test.

^b^IM: intramuscular.

^c^PORT: port-a-cath catheter system.

^d^IV: intravenous.

^e^IT: intrathecal.

^f^BMA: bone marrow aspiration.

^g^BT: blood transfusion.

The correction will appear in the online version of the paper on the JMIR Publications website on October 5, 2022, together with the publication of this correction notice. Because this was made after submission to PubMed, PubMed Central, and other full-text repositories, the corrected article has also been resubmitted to those repositories.

## Appendix

Multimedia Appendix 1 Originally published Table 3. Caregiver-reported invasive therapies.

