# Supplementary material for: Development of a Therapeutic Video Game With the MDA Framework to Decrease Anxiety in Preschool-Aged Children With Acute Lymphoblastic Leukemia: Mixed Methods Approach
Source: JMIR Serious Games. 2022 Aug 22;10(3):e37079. doi: 10.2196/37079 (PMC9446132; doi:10.2196/37079)

Stage(weeks)
Route x Dosages (Drugs, Schedule)

Drugs Information			
Abbrevation	Full Name	Route	Frequency
6-MP	6-mercaptopurine	PO	QD
ASP	L-asparaginase	IM	TIW
Ara-C	Cytarabine	IV	QD
Cyclo	Cyclophosphmide	IV	QW 6 hrs, Q12H x2 day
DEX	Dexamethasone	PO	TID
EPI	Epirubicin	IV	QW
VP-16	Etoposide	IV	QD
MTX	Methotrexate	IV	QD
PRE	Prednisone	PO	TID
VCR	Vincristine	IV	QW
TIT	Methotrexate + Hydrocortisone + Ara-C(MHA)	IT	QW

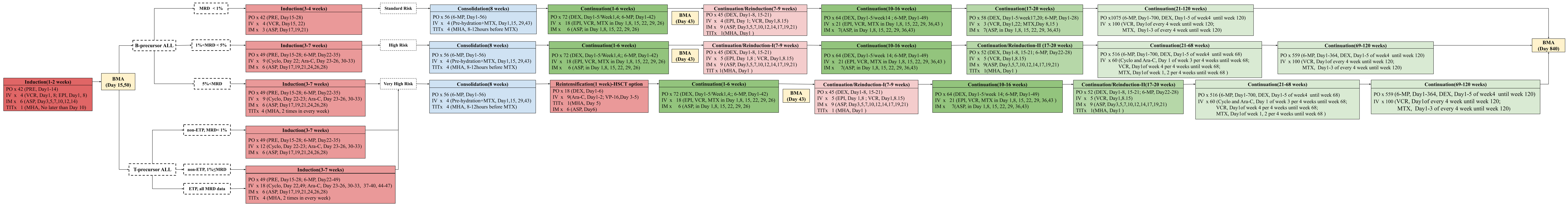

Supplement: Multimedia Appendix 1 [file games_v10i3e37079_app1.pdf]
