# Supplementary material for: Development of a Therapeutic Video Game With the MDA Framework to Decrease Anxiety in Preschool-Aged Children With Acute Lymphoblastic Leukemia: Mixed Methods Approach
Source: JMIR Serious Games. 2022 Aug 22;10(3):e37079. doi: 10.2196/37079 (PMC9446132; doi:10.2196/37079)

每日任務：抽骨髓
(Daily task) (BMA)

用物：棉花棒 (Supplies) (swab stick)

無菌布

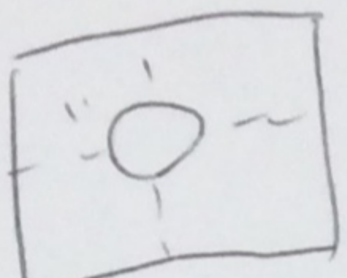

(Sterilized hole drape)

骨髓針 (BMA needle)

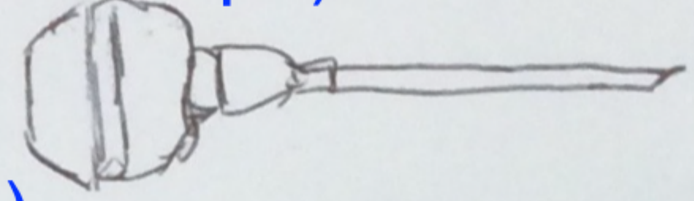

上網搜尋：骨髓穿刺針 (Searching BMA needle on line)

空針 (Syringe)

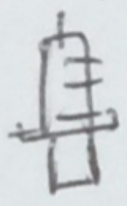

膠布 (Tape)

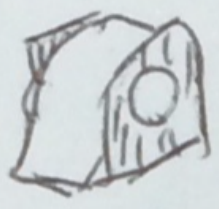

上網搜尋：3M透氣膠布 (Searching tapes on line)

~~大字~~

步驟：(Procedures)

1、點棉棒進行消毒 (Sterilization with swab stick)

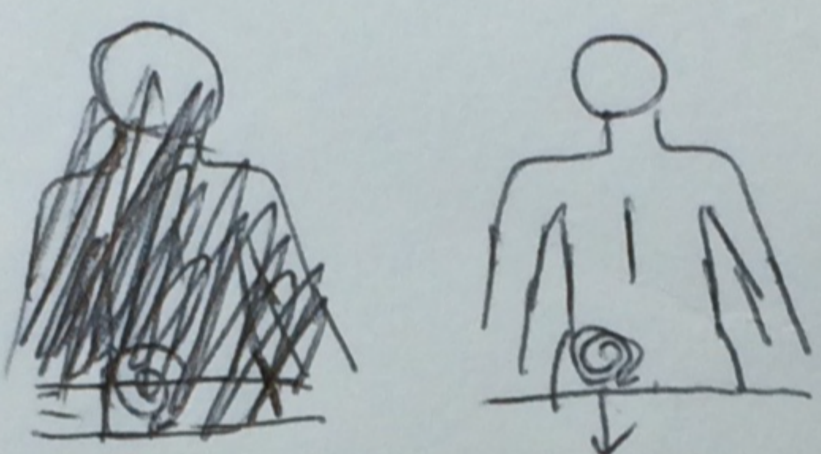

2、無菌布：畫面上出現布 (Covering with hole)

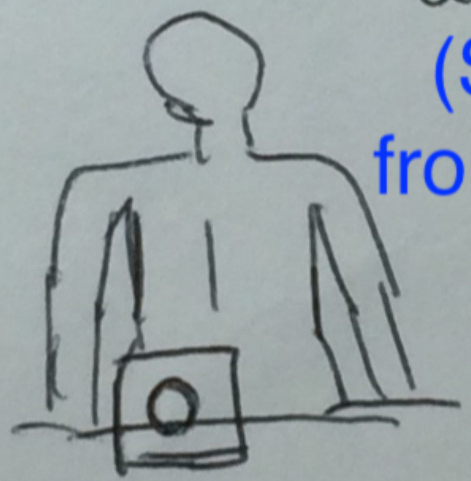

3、點選骨穿針，出現針入皮膚畫面 (Inserting BMA needle)

4、點選空針，出現抽骨髓畫面 (Collecting bone marrow)

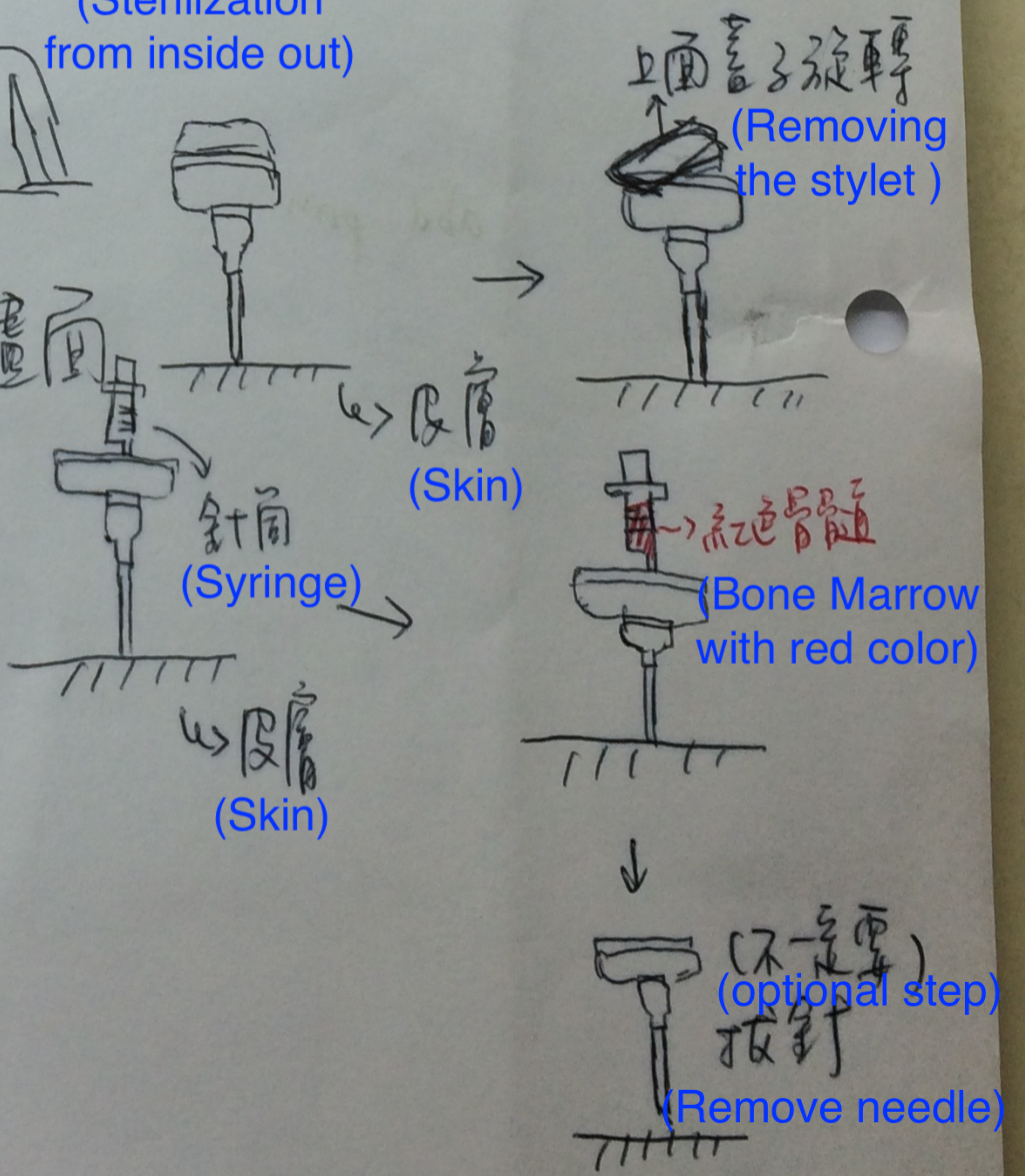

5、點選膠布：(Wound cover with gauze and tape)

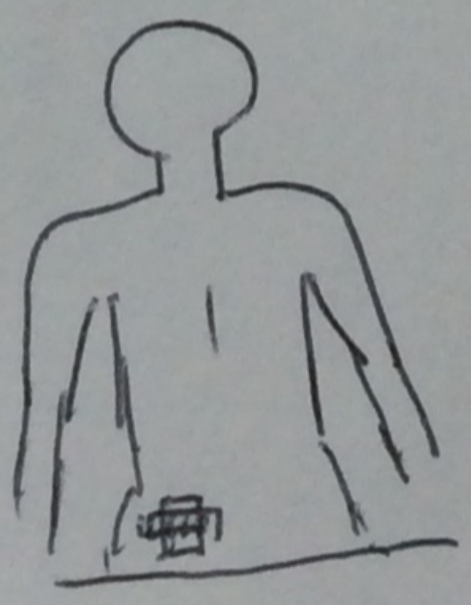

起始畫面 (Initial interface)

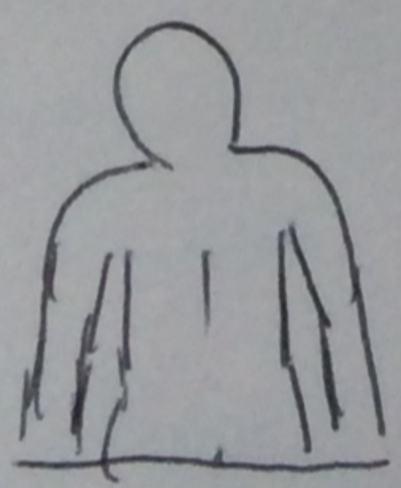

Supplement: Multimedia Appendix 2 [file games_v10i3e37079_app2.pdf]
