# Supplementary material for: Development of a Therapeutic Video Game With the MDA Framework to Decrease Anxiety in Preschool-Aged Children With Acute Lymphoblastic Leukemia: Mixed Methods Approach
Source: JMIR Serious Games. 2022 Aug 22;10(3):e37079. doi: 10.2196/37079 (PMC9446132; doi:10.2196/37079)
Supplement: Multimedia Appendix 3 [file games_v10i3e37079_app3.pdf]

CONSORT-EHEALTH (V 1.6.1) - Submission/Publication Form

The CONSORT-EHEALTH checklist is intended for authors of randomized trials evaluating web-based and Internet-based applications/interventions, including mobile interventions, electronic games (incl multiplayer games), social media, certain telehealth applications, and other interactive and/or networked electronic applications. Some of the items (e.g. all subitems under item 5 - description of the intervention) may also be applicable for other study designs.

The goal of the CONSORT EHEALTH checklist and guideline is to be
a) a guide for reporting for authors of RCTs,
b) to form a basis for appraisal of an ehealth trial (in terms of validity)

CONSORT-EHEALTH items/subitems are MANDATORY reporting items for studies published in the Journal of Medical Internet Research and other journals / scientific societies endorsing the checklist.

Items numbered 1., 2., 3., 4a., 4b etc are original CONSORT or CONSORT-NPT (non-pharmacologic treatment) items.
Items with Roman numerals (i., ii, iii, iv etc.) are CONSORT-EHEALTH extensions/clarifications.

As the CONSORT-EHEALTH checklist is still considered in a formative stage, we would ask that you also RATE ON A SCALE OF 1-5 how important/useful you feel each item is FOR THE PURPOSE OF THE CHECKLIST and reporting guideline (optional).

Mandatory reporting items are marked with a red *.
In the textboxes, either copy & paste the relevant sections from your manuscript into this form - please include any quotes from your manuscript in QUOTATION MARKS,
or answer directly by providing additional information not in the manuscript, or elaborating on why the item was not relevant for this study.

YOUR ANSWERS WILL BE PUBLISHED AS A SUPPLEMENTARY FILE TO YOUR PUBLICATION IN JMIR AND ARE CONSIDERED PART OF YOUR PUBLICATION (IF ACCEPTED).
Please fill in these questions diligently. Information will not be copyedited, so please use proper spelling and grammar, use correct capitalization, and avoid abbreviations.

DO NOT FORGET TO SAVE AS PDF _AND_ CLICK THE SUBMIT BUTTON SO YOUR ANSWERS ARE IN OUR DATABASE !!!

Citation Suggestion (if you append the pdf as Appendix we suggest to cite this paper in the caption):
Eysenbach G, CONSORT-EHEALTH Group
CONSORT-EHEALTH: Improving and Standardizing Evaluation Reports of Web-based and Mobile Health Interventions
J Med Internet Res 2011;13(4):e126

URL: http://www.jmir.org/2011/4/e126/
doi: 10.2196/jmir.1923
PMID: 22209829

evidta@gmail.com (未分享) 切換帳戶

已儲存草稿

*必填

Your name *

First Last

I-Ching Hou

Primary Affiliation (short), City, Country *

University of Toronto, Toronto, Canada

University of Yang Ming Chiao Tung University,

Your e-mail address *

abc@gmail.com

evita@nycu.edu.tw

Title of your manuscript *

Provide the (draft) title of your manuscript.

Developing the Therapeutic Video Game with MDA Framework to Decrease Anxiety for Pre-school Children with Acute Lymphoblastic Leukemia: Mix-method Approaches

Name of your App/Software/Intervention *

If there is a short and a long/alternate name, write the short name first and add the long name in brackets.

Therapeutic Video Games (name as “saving th

Evaluated Version (if any)

e.g. "V1", "Release 2017-03-01", "Version 2.0.27913"

Version 1

Language(s) *

What language is the intervention/app in? If multiple languages are available, separate by comma (e.g. "English, French")

Traditional Chinese

URL of your Intervention Website or App

e.g. a direct link to the mobile app on app in appstore (itunes, Google Play), or URL of the website. If the intervention is a DVD or hardware, you can also link to an Amazon page.

http://child.pctech.tw/home/login/1

URL of an image/screenshot (optional)

您的回答

Accessibility *

Can an enduser access the intervention presently?

- (selected) access is free and open
- access only for special usergroups, not open
- access is open to everyone, but requires payment/subscription/in-app purchases
- app/intervention no longer accessible
- 其他：

Primary Medical Indication/Disease/Condition *

e.g. "Stress", "Diabetes", or define the target group in brackets after the condition, e.g. "Autism (Parents of children with)", "Alzheimers (Informal Caregivers of)"

Acute Lymphoblastic Leukemia

Primary Outcomes measured in trial *

comma-separated list of primary outcomes reported in the trial

Anxiety response with facial ranking scale

Secondary/other outcomes

Are there any other outcomes the intervention is expected to affect?

No any other outcomes the intervention is expected to affect

Recommended "Dose" *

What do the instructions for users say on how often the app should be used?

- ○ Approximately Daily
- ○ Approximately Weekly
- ○ Approximately Monthly
- ○ Approximately Yearly
- ◉ "as needed"
- ○ 其他：

Approx. Percentage of Users (starters) still using the app as recommended after 3 months *

- ○ unknown / not evaluated
- ○ 0-10%
- ○ 11-20%
- ◉ 21-30%
- ○ 31-40%
- ○ 41-50%
- ○ 51-60%
- ○ 61-70%
- ○ 71%-80%
- ○ 81-90%
- ○ 91-100%
- ○ 其他：

Overall, was the app/intervention effective? *

- ○ yes: all primary outcomes were significantly better in intervention group vs control
- ◉ partly: SOME primary outcomes were significantly better in intervention group vs control
- ○ no statistically significant difference between control and intervention
- ○ potentially harmful: control was significantly better than intervention in one or more outcomes
- ○ inconclusive: more research is needed
- ○ 其他：

Article Preparation Status/Stage *

At which stage in your article preparation are you currently (at the time you fill in this form)

- ○ not submitted yet - in early draft status
- ○ not submitted yet - in late draft status, just before submission
- ○ submitted to a journal but not reviewed yet
- ○ submitted to a journal and after receiving initial reviewer comments
- ◉ submitted to a journal and accepted, but not published yet
- ○ published
- ○ 其他：

Journal *

If you already know where you will submit this paper (or if it is already submitted), please provide the journal name (if it is not JMIR, provide the journal name under "other")

- ○ not submitted yet / unclear where I will submit this
- ○ Journal of Medical Internet Research (JMIR)
- ○ JMIR mHealth and UHealth
- ◉ JMIR Serious Games
- ○ JMIR Mental Health
- ○ JMIR Public Health
- ○ JMIR Formative Research
- ○ Other JMIR sister journal
- ○ 其他：

Is this a full powered effectiveness trial or a pilot/feasibility trial? *

- ◉ Pilot/feasibility
- ○ Fully powered

Manuscript tracking number *

If this is a JMIR submission, please provide the manuscript tracking number under "other" (The ms tracking number can be found in the submission acknowledgement email, or when you login as author in JMIR. If the paper is already published in JMIR, then the ms tracking number is the four-digit number at the end of the DOI, to be found at the bottom of each published article in JMIR)

- ○ no ms number (yet) / not (yet) submitted to / published in JMIR
- ◉ 其他： ms# 37079

TITLE AND ABSTRACT

1a) TITLE: Identification as a randomized trial in the title

1a) Does your paper address CONSORT item 1a? *

I.e does the title contain the phrase "Randomized Controlled Trial"? (if not, explain the reason under "other")

- ○ yes
- ◉ 其他： this study included multiple approaches and included a randomized t

1a-i) Identify the mode of delivery in the title

Identify the mode of delivery. Preferably use “web-based” and/or “mobile” and/or “electronic game” in the title. Avoid ambiguous terms like “online”, “virtual”, “interactive”. Use “Internet-based” only if Intervention includes non-web-based Internet components (e.g. email), use “computer-based” or “electronic” only if offline products are used. Use “virtual” only in the context of “virtual reality” (3-D worlds). Use “online” only in the context of “online support groups”. Complement or substitute product names with broader terms for the class of products (such as “mobile” or “smart phone” instead of “iphone”), especially if the application runs on different platforms.

	1	2	3	4	5	
subitem not at all important	○	○	○	○	◉	essential

清除選取的項目

Does your paper address subitem 1a-i? *

Copy and paste relevant sections from manuscript title (include quotes in quotation marks "like this" to indicate direct quotes from your manuscript), or elaborate on this item by providing additional information not in the ms, or briefly explain why the item is not applicable/relevant for your study

Developing the Therapeutic “Video Game” with MDA Framework to Decrease Anxiety for Pre-school Children with Acute Lymphoblastic Leukemia: Mix-method Approaches

1a-ii) Non-web-based components or important co-interventions in title

Mention non-web-based components or important co-interventions in title, if any (e.g., “with telephone support”).

	1	2	3	4	5	
subitem not at all important	◉	○	○	○	○	essential

清除選取的項目

Does your paper address subitem 1a-ii?

Copy and paste relevant sections from manuscript title (include quotes in quotation marks "like this" to indicate direct quotes from your manuscript), or elaborate on this item by providing additional information not in the ms, or briefly explain why the item is not applicable/relevant for your study

We do not include a co-intervention in this work.

1a-iii) Primary condition or target group in the title

Mention primary condition or target group in the title, if any (e.g., “for children with Type I Diabetes”) Example: A Web-based and Mobile Intervention with Telephone Support for Children with Type I Diabetes: Randomized Controlled Trial

	1	2	3	4	5	
subitem not at all important	○	○	○	○	◉	essential

清除選取的項目

Does your paper address subitem 1a-iii? *

Copy and paste relevant sections from manuscript title (include quotes in quotation marks "like this" to indicate direct quotes from your manuscript), or elaborate on this item by providing additional information not in the ms, or briefly explain why the item is not applicable/relevant for your study

Developing the Therapeutic Video Game with MDA Framework to Decrease Anxiety for Pre-school Children with “Acute Lymphoblastic Leukemia”: Mix-method Approaches

1b) ABSTRACT: Structured summary of trial design, methods, results, and conclusions

NPT extension: Description of experimental treatment, comparator, care providers, centers, and blinding status.

1b-i) Key features/functionalities/components of the intervention and comparator in the METHODS section of the ABSTRACT

Mention key features/functionalities/components of the intervention and comparator in the abstract. If possible, also mention theories and principles used for designing the site. Keep in mind the needs of systematic reviewers and indexers by including important synonyms. (Note: Only report in the abstract what the main paper is reporting. If this information is missing from the main body of text, consider adding it)

	1	2	3	4	5	
subitem not at all important	○	○	○	○	◉	essential

清除選取的項目

Does your paper address subitem 1b-i? *

Copy and paste relevant sections from the manuscript abstract (include quotes in quotation marks "like this" to indicate direct quotes from your manuscript), or elaborate on this item by providing additional information not in the ms, or briefly explain why the item is not applicable/relevant for your study

“Eligible preschoolers with ALL were divided into experimental group and control group with ration of 1:1. Both group subjects received the same usual care and only experimental group used TVG.”

1b-ii) Level of human involvement in the METHODS section of the ABSTRACT

Clarify the level of human involvement in the abstract, e.g., use phrases like “fully automated” vs. “therapist/nurse/care provider/physician-assisted” (mention number and expertise of providers involved, if any). (Note: Only report in the abstract what the main paper is reporting. If this information is missing from the main body of text, consider adding it)

	1	2	3	4	5	
subitem not at all important	○	○	○	○	◉	essential

清除選取的項目

Does your paper address subitem 1b-ii?

Copy and paste relevant sections from the manuscript abstract (include quotes in quotation marks "like this" to indicate direct quotes from your manuscript), or elaborate on this item by providing additional information not in the ms, or briefly explain why the item is not applicable/relevant for your study

To evaluate the therapeutic anxiety reduction after “using TVG for six weeks with two-group stratified randomized controlled trial” in one medical center in northern Taiwan.

1b-iii) Open vs. closed, web-based (self-assessment) vs. face-to-face assessments in the METHODS section of the ABSTRACT

Mention how participants were recruited (online vs. offline), e.g., from an open access website or from a clinic or a closed online user group (closed usergroup trial), and clarify if this was a purely web-based trial, or there were face-to-face components (as part of the intervention or for assessment). Clearly say if outcomes were self-assessed through questionnaires (as common in web-based trials). Note: In traditional offline trials, an open trial (open-label trial) is a type of clinical trial in which both the researchers and participants know which treatment is being administered. To avoid confusion, use “blinded” or “unblinded” to indicated the level of blinding instead of “open”, as “open” in web-based trials usually refers to “open access” (i.e. participants can self-enrol). (Note: Only report in the abstract what the main paper is reporting. If this information is missing from the main body of text, consider adding it)

	1	2	3	4	5	
subitem not at all important	○	○	○	○	◉	essential

清除選取的項目

Does your paper address subitem 1b-iii?

Copy and paste relevant sections from the manuscript abstract (include quotes in quotation marks "like this" to indicate direct quotes from your manuscript), or elaborate on this item by providing additional information not in the ms, or briefly explain why the item is not applicable/relevant for your study

The children anxiety response was “reported” with facial ranking scale (FRS) by their family caregiver.

1b-iv) RESULTS section in abstract must contain use data

Report number of participants enrolled/assessed in each group, the use/uptake of the intervention (e.g., attrition/adherence metrics, use over time, number of logins etc.), in addition to primary/secondary outcomes. (Note: Only report in the abstract what the main paper is reporting. If this information is missing from the main body of text, consider adding it)

	1	2	3	4	5	
subitem not at all important	○	○	○	○	◉	essential

清除選取的項目

Does your paper address subitem 1b-iv?

Copy and paste relevant sections from the manuscript abstract (include quotes in quotation marks "like this" to indicate direct quotes from your manuscript), or elaborate on this item by providing additional information not in the ms, or briefly explain why the item is not applicable/relevant for your study

“A total of 15 participants were enrolled and randomly allocated to the experimental (n=7) or the control (n=8) groups.”

1b-v) CONCLUSIONS/DISCUSSION in abstract for negative trials

Conclusions/Discussions in abstract for negative trials: Discuss the primary outcome - if the trial is negative (primary outcome not changed), and the intervention was not used, discuss whether negative results are attributable to lack of uptake and discuss reasons. (Note: Only report in the abstract what the main paper is reporting. If this information is missing from the main body of text, consider adding it)

	1	2	3	4	5	
subitem not at all important	○	○	○	○	◉	essential

清除選取的項目

Does your paper address subitem 1b-v?

Copy and paste relevant sections from the manuscript abstract (include quotes in quotation marks "like this" to indicate direct quotes from your manuscript), or elaborate on this item by providing additional information not in the ms, or briefly explain why the item is not applicable/relevant for your study

This research provides some evidence for using a therapeutic video game to decrease their anxiety for ALL preschoolers in Taiwan.

INTRODUCTION

2a) In INTRODUCTION: Scientific background and explanation of rationale

2a-i) Problem and the type of system/solution

Describe the problem and the type of system/solution that is object of the study: intended as stand-alone intervention vs. incorporated in broader health care program? Intended for a particular patient population? Goals of the intervention, e.g., being more cost-effective to other interventions, replace or complement other solutions? (Note: Details about the intervention are provided in “Methods” under 5)

	1	2	3	4	5	
subitem not at all important	○	○	○	○	◉	essential

清除選取的項目

Does your paper address subitem 2a-i? *

Copy and paste relevant sections from the manuscript (include quotes in quotation marks "like this" to indicate direct quotes from your manuscript), or elaborate on this item by providing additional information not in the ms, or briefly explain why the item is not applicable/relevant for your study

“In order to reduce the therapy anxiety of sick children, many medical professionals used to cognitive/attentional distraction in the control of pediatric patients pre/during/post treatments [11, 12]. However, the cost of invisible companion time of medical staff has also increased, and the demand for clinical medical manpower has increased. With the popularization of electronic technology products such as computers, smart phones and tablets, in addition to the traditional approaches (e.g., drawing, role play, and toy operations). The game programs in portable electronic products have gradually become another aspect of children’s game activities. Many studies have applied video games or virtual reality on pediatric care [13-15]. However, this type of research is mostly limited to the use of general electronic games to divert attention, rather than specially designed therapeutic electronic games.”

2a-ii) Scientific background, rationale: What is known about the (type of) system

Scientific background, rationale: What is known about the (type of) system that is the object of the study (be sure to discuss the use of similar systems for other conditions/diagnoses, if appropiate), motivation for the study, i.e. what are the reasons for and what is the context for this specific study, from which stakeholder viewpoint is the study performed, potential impact of findings [2]. Briefly justify the choice of the comparator.

	1	2	3	4	5	
subitem not at all important	○	○	○	○	◉	essential

清除選取的項目

Does your paper address subitem 2a-ii? *

Copy and paste relevant sections from the manuscript (include quotes in quotation marks "like this" to indicate direct quotes from your manuscript), or elaborate on this item by providing additional information not in the ms, or briefly explain why the item is not applicable/relevant for your study

“With the popularization of electronic technology products such as computers, smart phones and tablets, in addition to the traditional approaches (e.g., drawing, role play, and toy operations). The game programs in portable electronic products have gradually become another aspect of children’s game activities. Many studies have applied video games or virtual reality on pediatric care [13-15].”

2b) In INTRODUCTION: Specific objectives or hypotheses

Does your paper address CONSORT subitem 2b? *

Copy and paste relevant sections from the manuscript (include quotes in quotation marks "like this" to indicate direct quotes from your manuscript), or elaborate on this item by providing additional information not in the ms, or briefly explain why the item is not applicable/relevant for your study

“the purpose of this study was to develop a video game that can be played by children with ALL in this age group based on the cognitive development and game patterns of preschool children, so as to understand the improvement of the anxiety response of therapeutic video games on children with acute lymphoblastic leukemia in preschool years.”

METHODS

3a) Description of trial design (such as parallel, factorial) including allocation ratio

Does your paper address CONSORT subitem 3a? *

Copy and paste relevant sections from the manuscript (include quotes in quotation marks "like this" to indicate direct quotes from your manuscript), or elaborate on this item by providing additional information not in the ms, or briefly explain why the item is not applicable/relevant for your study

“The TVG trial was a patient blinded, parallel group, stratified randomized controlled trial with six weeks follow-up evaluations.”
…
Participants were recruited by the researchers (DJ) and one trained research assistant in the pediatric hematology ward or outpatient clinic. The eligible patients were screened by the researcher (DJ) before the recruitment in the study setting according to the 2015-2016 Taiwan ALL incidence rate of each age and sex strata[3]. Then each age and sex strata of participants was randomly assigned 1:1 into one of two study groups (control or experimental). The randomization scheme was generated by using the web site: www.randomization.com for each strate.

3b) Important changes to methods after trial commencement (such as eligibility criteria), with reasons

Does your paper address CONSORT subitem 3b? *

Copy and paste relevant sections from the manuscript (include quotes in quotation marks "like this" to indicate direct quotes from your manuscript), or elaborate on this item by providing additional information not in the ms, or briefly explain why the item is not applicable/relevant for your study

No important changes to methods after trial commencement

3b-i) Bug fixes, Downtimes, Content Changes

Bug fixes, Downtimes, Content Changes: ehealth systems are often dynamic systems. A description of changes to methods therefore also includes important changes made on the intervention or comparator during the trial (e.g., major bug fixes or changes in the functionality or content) (5-iii) and other “unexpected events” that may have influenced study design such as staff changes, system failures/downtimes, etc. [2].

	1	2	3	4	5	
subitem not at all important	○	○	○	○	◉	essential

清除選取的項目

Does your paper address subitem 3b-i?

Copy and paste relevant sections from the manuscript (include quotes in quotation marks "like this" to indicate direct quotes from your manuscript), or elaborate on this item by providing additional information not in the ms, or briefly explain why the item is not applicable/relevant for your study

There were no bug fixes, downtimes, or content changes needed in this trial.

4a) Eligibility criteria for participants

Does your paper address CONSORT subitem 4a? *

Copy and paste relevant sections from the manuscript (include quotes in quotation marks "like this" to indicate direct quotes from your manuscript), or elaborate on this item by providing additional information not in the ms, or briefly explain why the item is not applicable/relevant for your study

“Preschoolers who met the following inclusion criteria were recruited: (1) diagnosis of acute lymphatic leukemia (ALL); (2) aged 3 to 5 years. Exclusion criteria were: (1) not underwent Taiwan Pediatric Oncology Group (TPOG) treatment guideline; (2) without Port-A insertion; (3) under peripheral blood stem cell transplantation treatment; (4) ALL recurrent; (5) diagnosis of mental retardation which TVG did not support their needs yet in this trial.”

4a-i) Computer / Internet literacy

Computer / Internet literacy is often an implicit “de facto” eligibility criterion - this should be explicitly clarified.

	1	2	3	4	5	
subitem not at all important	○	○	◉	○	○	essential

清除選取的項目

Does your paper address subitem 4a-i?

Copy and paste relevant sections from the manuscript (include quotes in quotation marks "like this" to indicate direct quotes from your manuscript), or elaborate on this item by providing additional information not in the ms, or briefly explain why the item is not applicable/relevant for your study

It has been clinically observed that the frequency of portable electronic products used by children of this age group is extremely high.

4a-ii) Open vs. closed, web-based vs. face-to-face assessments:

Open vs. closed, web-based vs. face-to-face assessments: Mention how participants were recruited (online vs. offline), e.g., from an open access website or from a clinic, and clarify if this was a purely web-based trial, or there were face-to-face components (as part of the intervention or for assessment), i.e., to what degree got the study team to know the participant. In online-only trials, clarify if participants were quasi-anonymous and whether having multiple identities was possible or whether technical or logistical measures (e.g., cookies, email confirmation, phone calls) were used to detect/prevent these.

	1	2	3	4	5	
subitem not at all important	○	○	○	○	◉	essential

清除選取的項目

Does your paper address subitem 4a-ii? *

Copy and paste relevant sections from the manuscript (include quotes in quotation marks "like this" to indicate direct quotes from your manuscript), or elaborate on this item by providing additional information not in the ms, or briefly explain why the item is not applicable/relevant for your study

“In this study, the children therapeutic anxiety response was reported by the participants’ family caregivers through web form which installed into their mobile phone when enrolled. The children anxiety response report web form consisted of four items: invasive therapy date, invasive therapies, face rating scale (FRS) and other response. The second item: invasive therapies included six activities (e.g., spanking shot; chemical medication administration; intrathecal injection; bone marrow aspiration, Port-A insertion, blood transfusion). The third item: FRS included 10 faces and their numbers which smile face with mouth open (e.g., number 1) indicate no cry at all; redness face with two tears (e.g., number 10) indicate severe cry. The fourth item: other response allowed family caregivers to free note any observation of the participants. When the participant completed any one of six invasive therapies, the family caregiver would help them to point out on the FRS that could express their real feeling during that therapy. The average time that care giver required to complete the report was less than 5 minutes without any assistance.”

4a-iii) Information giving during recruitment

Information given during recruitment. Specify how participants were briefed for recruitment and in the informed consent procedures (e.g., publish the informed consent documentation as appendix, see also item X26), as this information may have an effect on user self-selection, user expectation and may also bias results.

	1	2	3	4	5	
subitem not at all important	○	○	○	○	◉	essential

清除選取的項目

Does your paper address subitem 4a-iii?

Copy and paste relevant sections from the manuscript (include quotes in quotation marks "like this" to indicate direct quotes from your manuscript), or elaborate on this item by providing additional information not in the ms, or briefly explain why the item is not applicable/relevant for your study

"Participation in the study was voluntary. The eligible participants were provided two different informed consents according to their assigned groups. The consents included the same information on the participants' cry scale and resist behavior data collection. Only the experiment groups' consents provide the briefing information of the TVG."

4b) Settings and locations where the data were collected

Does your paper address CONSORT subitem 4b? *

Copy and paste relevant sections from the manuscript (include quotes in quotation marks "like this" to indicate direct quotes from your manuscript), or elaborate on this item by providing additional information not in the ms, or briefly explain why the item is not applicable/relevant for your study

"The study site was the medical center (e.g., National Taiwan University Hospital) in northern Taiwan. Every patient in the study site received the same verbal comfort from nurses before/ during the process of invasive therapy. Some attention shifting skill (e.g., watching cartoon video, listening to nursery rhymes & kid's songs, providing comfort toys etc.) would also be adopted but non-standardized by their caregiver and professionals to facilitate the procedure of therapy."

4b-i) Report if outcomes were (self-)assessed through online questionnaires

Clearly report if outcomes were (self-)assessed through online questionnaires (as common in web-based trials) or otherwise.

	1	2	3	4	5	
subitem not at all important	○	○	○	○	◉	essential

清除選取的項目

Does your paper address subitem 4b-i? *

Copy and paste relevant sections from the manuscript (include quotes in quotation marks "like this" to indicate direct quotes from your manuscript), or elaborate on this item by providing additional information not in the ms, or briefly explain why the item is not applicable/relevant for your study

“In this study, the children therapeutic anxiety response was reported by the participants' family caregivers through web form which installed into their mobile phone when enrolled. The children anxiety response report web form consisted of four items: invasive therapy date, invasive therapies, face rating scale (FRS) and other response. The second item: invasive therapies included six activities (e.g., spanking shot; chemical medication administration; intrathecal injection; bone marrow aspiration, Port-A insertion, blood transfusion). The third item: FRS included 10 faces and their numbers which smile face with mouth open (e.g., number 1) indicate no cry at all; redness face with two tears (e.g., number 10) indicate severe cry. The fourth item: other response allowed family caregivers to free note any observation of the participants. When the participant completed any one of six invasive therapies, the family caregiver would help them to point out on the FRS that could express their real feeling during that therapy. The average time that care giver required to complete the report was less than 5 minutes without any assistance.”

4b-ii) Report how institutional affiliations are displayed

Report how institutional affiliations are displayed to potential participants [on ehealth media], as affiliations with prestigious hospitals or universities may affect volunteer rates, use, and reactions with regards to an intervention.(Not a required item – describe only if this may bias results)

	1	2	3	4	5	
subitem not at all important	○	○	○	○	◉	essential

清除選取的項目

Does your paper address subitem 4b-ii?

Copy and paste relevant sections from the manuscript (include quotes in quotation marks "like this" to indicate direct quotes from your manuscript), or elaborate on this item by providing additional information not in the ms, or briefly explain why the item is not applicable/relevant for your study

This study followed the ethical principles of the Declaration of Helsinki [18] and approved by the Institute of Review Board in National Taiwan University Hospital (IRB No. 201705014RINC) in Taiwan before trial started.
…
Data collection was from March, 2018 to April in 2020 by the study team. Patients were blinded whether they were in the experimental or control group. The demographics data (e.g., age, sex, diagnosis, risk classifications, treatment stage) of the eligible participants were collected through the electronic health record in the study site.

5) The interventions for each group with sufficient details to allow replication, including how and when they were actually administered

5-i) Mention names, credential, affiliations of the developers, sponsors, and owners

Mention names, credential, affiliations of the developers, sponsors, and owners [6] (if authors/evaluators are owners or developer of the software, this needs to be declared in a “Conflict of interest” section or mentioned elsewhere in the manuscript).

	1	2	3	4	5	
subitem not at all important	○	○	○	○	◉	essential

清除選取的項目

Does your paper address subitem 5-i?

Copy and paste relevant sections from the manuscript (include quotes in quotation marks "like this" to indicate direct quotes from your manuscript), or elaborate on this item by providing additional information not in the ms, or briefly explain why the item is not applicable/relevant for your study

To decreasing the therapeutic anxiety for preschoolers with ALL, the research team cooperated with Taiwan video game technology company to develop the TVG.

5-ii) Describe the history/development process

Describe the history/development process of the application and previous formative evaluations (e.g., focus groups, usability testing), as these will have an impact on adoption/use rates and help with interpreting results.

	1	2	3	4	5	
subitem not at all important	○	○	○	○	●	essential

清除選取的項目

Does your paper address subitem 5-ii?

Copy and paste relevant sections from the manuscript (include quotes in quotation marks "like this" to indicate direct quotes from your manuscript), or elaborate on this item by providing additional information not in the ms, or briefly explain why the item is not applicable/relevant for your study

“the research team cooperated with Taiwan video game technology company to develop the TVG. The group meetings were held to make the consensus on the MDA framework [16]. The game designer (DJ) who was also register nurse in the pediatric oncology ward based on the knowledge of the preschoolers’ cognitive-developmental stage (e.g., preoperational) that the children with three to five years old show egocentrism, anthropomorphism, thinking without logic and lacking conservation [17]. Thus, to facilitating aesthetic when playing the game, the design of TVG would avoid the mechanic/dynamic with complex logical (e.g., calculation) or conservation (e.g., comparison) but to provide the anthropomorphic role, easy and repeatable activities, low challenge tasks etc.
With the cooperation of TVG development, consensus meetings were usually held (e.g., once per week). The team first focused on the aesthetics expectations and dynamic features and then defined the mechanic data representations and algorithms. When the MDA framework was conceptually saturated and logically feasible, the game designer (DJ) provided user interface manuscript including procedures of invasive therapies and required medical supplies (see Supplementary file 2) to the visual designer and the programmer to facilitate them on designing the game for ALL preschoolers.”

5-iii) Revisions and updating

Revisions and updating. Clearly mention the date and/or version number of the application/intervention (and comparator, if applicable) evaluated, or describe whether the intervention underwent major changes during the evaluation process, or whether the development and/or content was “frozen” during the trial. Describe dynamic components such as news feeds or changing content which may have an impact on the replicability of the intervention (for unexpected events see item 3b).

	1	2	3	4	5	
subitem not at all important	○	○	○	○	●	essential

清除選取的項目

Does your paper address subitem 5-iii?

Copy and paste relevant sections from the manuscript (include quotes in quotation marks "like this" to indicate direct quotes from your manuscript), or elaborate on this item by providing additional information not in the ms, or briefly explain why the item is not applicable/relevant for your study

“To assess the reliability of TVG, three certified children art therapists were invited to preview the TVG. Then they completed one 12-item instrument which contain usefulness (9 items), trust (2 items) structured questions with five-point Likert scale (5: strongly agree; 1: strongly disagree) and narrative feedback (1 item) open question. The descriptive analysis of eleven structured items, content analysis of one narrative feedback item and internal consistence reliability (Cronbach α level) of three therapists were performed to evaluate the usefulness and trust of the TVG. The results were also used to optimize the TVG before phase 3.”
...
“All the intervention group participants received the same TVG (e.g., frozen version) and there were no any technical problems associated with the TVG during the trial.”

5-iv) Quality assurance methods

Provide information on quality assurance methods to ensure accuracy and quality of information provided [1], if applicable.

	1	2	3	4	5	
subitem not at all important	○	○	○	○	◉	essential

清除選取的項目

Does your paper address subitem 5-iv?

Copy and paste relevant sections from the manuscript (include quotes in quotation marks "like this" to indicate direct quotes from your manuscript), or elaborate on this item by providing additional information not in the ms, or briefly explain why the item is not applicable/relevant for your study

“To assess the reliability of TVG, three certified children art therapists were invited to preview the TVG. Then they completed one 12-item instrument which contain usefulness (9 items), trust (2 items) structured questions with five-point Likert scale (5: strongly agree; 1: strongly disagree) and narrative feedback (1 item) open question. The descriptive analysis of eleven structured items, content analysis of one narrative feedback item and internal consistence reliability (Cronbach α level) of three therapists were performed to evaluate the usefulness and trust of the TVG. The results were also used to optimize the TVG before phase 3.”

5-v) Ensure replicability by publishing the source code, and/or providing screenshots/screen-capture video, and/or providing flowcharts of the algorithms used

Ensure replicability by publishing the source code, and/or providing screenshots/screen-capture video, and/or providing flowcharts of the algorithms used. Replicability (i.e., other researchers should in principle be able to replicate the study) is a hallmark of scientific reporting.

	1	2	3	4	5	
subitem not at all important	○	○	○	○	◉	essential

清除選取的項目

Does your paper address subitem 5-v?

Copy and paste relevant sections from the manuscript (include quotes in quotation marks "like this" to indicate direct quotes from your manuscript), or elaborate on this item by providing additional information not in the ms, or briefly explain why the item is not applicable/relevant for your study

After the guideline of MDA framework (see Figure 1), the TVG (name as “saving the planet of animal”) was developed with webpage technology and could be play on the website.
...
“Taking the BMA task as example (see Table 2), the player had the character to follow the task rule by executing preparation, sterilization, covering the sterile area with hole drape, inserting BMA needle, collecting bone marrow, and wound cover with gauze.”

5-vi) Digital preservation

Digital preservation: Provide the URL of the application, but as the intervention is likely to change or disappear over the course of the years; also make sure the intervention is archived (Internet Archive, webcitation.org, and/or publishing the source code or screenshots/videos alongside the article). As pages behind login screens cannot be archived, consider creating demo pages which are accessible without login.

	1	2	3	4	5	
subitem not at all important	○	○	◉	○	○	essential

清除選取的項目

Does your paper address subitem 5-vi?

Copy and paste relevant sections from the manuscript (include quotes in quotation marks "like this" to indicate direct quotes from your manuscript), or elaborate on this item by providing additional information not in the ms, or briefly explain why the item is not applicable/relevant for your study

After the guideline of MDA framework (see Figure 1), the TVG (name as “saving the planet of animal”) was developed with webpage technology and could be play on the website.
...
“Taking the BMA task as example (see Table 2), the player had the character to follow the task rule by executing preparation, sterilization, covering the sterile area with hole drape, inserting BMA needle, collecting bone marrow, and wound cover with gauze.”

5-vii) Access

Access: Describe how participants accessed the application, in what setting/context, if they had to pay (or were paid) or not, whether they had to be a member of specific group. If known, describe how participants obtained “access to the platform and Internet” [1]. To ensure access for editors/reviewers/readers, consider to provide a “backdoor” login account or demo mode for reviewers/readers to explore the application (also important for archiving purposes, see vi).

	1	2	3	4	5	
subitem not at all important	○	○	◉	○	○	essential

清除選取的項目

Does your paper address subitem 5-vii? *

Copy and paste relevant sections from the manuscript (include quotes in quotation marks "like this" to indicate direct quotes from your manuscript), or elaborate on this item by providing additional information not in the ms, or briefly explain why the item is not applicable/relevant for your study

After the guideline of MDA framework (see Figure 1), the TVG (name as “saving the planet of animal”) was developed with webpage technology and could be play on the website.
…
“Taking the BMA task as example (see Table 2), the player had the character to follow the task rule by executing preparation, sterilization, covering the sterile area with hole drape, inserting BMA needle, collecting bone marrow, and wound cover with gauze.”

5-viii) Mode of delivery, features/functionalities/components of the intervention and comparator, and the theoretical framework

Describe mode of delivery, features/functionalities/components of the intervention and comparator, and the theoretical framework [6] used to design them (instructional strategy [1], behaviour change techniques, persuasive features, etc., see e.g., [7, 8] for terminology). This includes an in-depth description of the content (including where it is coming from and who developed it) [1],” whether [and how] it is tailored to individual circumstances and allows users to track their progress and receive feedback” [6]. This also includes a description of communication delivery channels and – if computer-mediated communication is a component – whether communication was synchronous or asynchronous [6]. It also includes information on presentation strategies [1], including page design principles, average amount of text on pages, presence of hyperlinks to other resources, etc. [1].

	1	2	3	4	5	
subitem not at all important	○	○	○	○	◉	essential

清除選取的項目

Does your paper address subitem 5-viii? *

Copy and paste relevant sections from the manuscript (include quotes in quotation marks "like this" to indicate direct quotes from your manuscript), or elaborate on this item by providing additional information not in the ms, or briefly explain why the item is not applicable/relevant for your study

The MDA framework for game design and research was published by Roubin Hunickes, Marc LeBlanc and Robert Zubek at 2004[16]. Mechanics describes the particular components of the game, at the level of data representation and algorithms (e.g., rules). Dynamics describes the run-time behavior of the mechanics acting on player inputs and each other's outputs over time. Aesthetics describes the desirable emotional responses evoked in the player, when player interacts with the game system. These emotional responses are including but are not limited to the taxonomy of sensation (e.g., game as sense-pleasure), fantasy (e.g., game as make-believe), narrative (e.g., game as drama), challenge (e.g., game as obstacle), fellowship (e.g., game as social framework), discovery (e.g., game as uncharted territory), expression (e.g., game as self-discovery), submission (e.g., game as pastime). Each component of MDA framework causally linked by the game designers and provided the players pleasure/fun perceptions [16]. When designing video game for sick preschoolers to decrease their therapeutic anxiety, the MDA framework could be the guideline for the game designer

....

After the guideline of MDA framework (see Figure 1), the TVG (name as "saving the planet of animal") was developed with webpage technology and could be play on the website.

With regard to mechanic components, a total of six rules which included one or several data representations and algorithms to support the dynamic of TVG. The "pause" rule was designed to remind the players break appropriately. The "full, happy and energy" rules was designed to make the players kept well habits in their real life by eating food, playing the toy and resting after playing the TVG. The "task" rule was designed to guide the players to familiar with the procedure of their invasive therapies. The TVG contained encourage voice (e.g., "good job!"), image (e.g., character show off with firework) when player complete any task as the positive feedback. The "money" rule was designed to encourage the players to keep overcoming more tasks. Table 1 showed the partial snapshot of the mechanics.

5-ix) Describe use parameters

Describe use parameters (e.g., intended "doses" and optimal timing for use). Clarify what instructions or recommendations were given to the user, e.g., regarding timing, frequency, heaviness of use, if any, or was the intervention used ad libitum.

	1	2	3	4	5	
subitem not at all important	○	○	○	◉	○	essential

清除選取的項目

Does your paper address subitem 5-ix?

Copy and paste relevant sections from the manuscript (include quotes in quotation marks "like this" to indicate direct quotes from your manuscript), or elaborate on this item by providing additional information not in the ms, or briefly explain why the item is not applicable/relevant for your study

"Each experimental participant could play the TVG in any time as needed (e.g., no any prompts/reminders from the study team)."

5-x) Clarify the level of human involvement

Clarify the level of human involvement (care providers or health professionals, also technical assistance) in the e-intervention or as co-intervention (detail number and expertise of professionals involved, if any, as well as "type of assistance offered, the timing and frequency of the support, how it is initiated, and the medium by which the assistance is delivered". It may be necessary to distinguish between the level of human involvement required for the trial, and the level of human involvement required for a routine application outside of a RCT setting (discuss under item 21 – generalizability).

	1	2	3	4	5	
subitem not at all important	○	○	○	○	◉	essential

清除選取的項目

Does your paper address subitem 5-x?

Copy and paste relevant sections from the manuscript (include quotes in quotation marks "like this" to indicate direct quotes from your manuscript), or elaborate on this item by providing additional information not in the ms, or briefly explain why the item is not applicable/relevant for your study

"Data collection was from March, 2018 to April in 2020 by the study team. Patients were blinded whether they were in the experimental or control group. The demographics data (e.g., age, sex, diagnosis, risk classifications, treatment stage) of the eligible participants were collected through the electronic health record in the study site. The TVG was only installed on the experimental participants' primary caregivers' smart phones and they were taught how to use it."

5-xi) Report any prompts/reminders used

Report any prompts/reminders used: Clarify if there were prompts (letters, emails, phone calls, SMS) to use the application, what triggered them, frequency etc. It may be necessary to distinguish between the level of prompts/reminders required for the trial, and the level of prompts/reminders for a routine application outside of a RCT setting (discuss under item 21 – generalizability).

	1	2	3	4	5	
subitem not at all important	○	○	○	○	◉	essential

清除選取的項目

Does your paper address subitem 5-xi? *

Copy and paste relevant sections from the manuscript (include quotes in quotation marks "like this" to indicate direct quotes from your manuscript), or elaborate on this item by providing additional information not in the ms, or briefly explain why the item is not applicable/relevant for your study

"Each experimental participant could play the TVG in any time as needed (e.g., no any prompts/reminders from the study team)."

5-xii) Describe any co-interventions (incl. training/support)

Describe any co-interventions (incl. training/support): Clearly state any interventions that are provided in addition to the targeted eHealth intervention, as ehealth intervention may not be designed as stand-alone intervention. This includes training sessions and support [1]. It may be necessary to distinguish between the level of training required for the trial, and the level of training for a routine application outside of a RCT setting (discuss under item 21 – generalizability.

	1	2	3	4	5	
subitem not at all important	○	◉	○	○	○	essential

清除選取的項目

Does your paper address subitem 5-xii? *

Copy and paste relevant sections from the manuscript (include quotes in quotation marks "like this" to indicate direct quotes from your manuscript), or elaborate on this item by providing additional information not in the ms, or briefly explain why the item is not applicable/relevant for your study

No co-interventions were used.

6a) Completely defined pre-specified primary and secondary outcome measures, including how and when they were assessed

Does your paper address CONSORT subitem 6a? *

Copy and paste relevant sections from the manuscript (include quotes in quotation marks "like this" to indicate direct quotes from your manuscript), or elaborate on this item by providing additional information not in the ms, or briefly explain why the item is not applicable/relevant for your study

“In this study, the children therapeutic anxiety response was reported by the participants’ family caregivers through web form which installed into their mobile phone when enrolled. The children anxiety response report web form consisted of four items: invasive therapy date, invasive therapies, face rating scale (FRS) and other response. The second item: invasive therapies included six activities (e.g., spanking shot; chemical medication administration; intrathecal injection; bone marrow aspiration, Port-A insertion, blood transfusion). The third item: FRS included 10 faces and their numbers which smile face with mouth open (e.g., number 1) indicate no cry at all; redness face with two tears (e.g., number 10) indicate severe cry. The fourth item: other response allowed family caregivers to free note any observation of the participants. When the participant completed any one of six invasive therapies, the family caregiver would help them to point out on the FRS that could express their real feeling during that therapy. The average time that care giver required to complete the report was less than 5 minutes without any assistance.”

6a-i) Online questionnaires: describe if they were validated for online use and apply CHERRIES items to describe how the questionnaires were designed/deployed

If outcomes were obtained through online questionnaires, describe if they were validated for online use and apply CHERRIES items to describe how the questionnaires were designed/deployed [9].

	1	2	3	4	5	
subitem not at all important	○	○	○	○	◉	essential

清除選取的項目

Does your paper address subitem 6a-i?

Copy and paste relevant sections from manuscript text

“According to the participants’ FRS reports, the internal consistence reliability of FRS was 0.52 (e.g., Cronbach’s Alpha) which indicated the acceptable reliability [19].”

6a-ii) Describe whether and how “use” (including intensity of use/dosage) was defined/measured/monitored

Describe whether and how “use” (including intensity of use/dosage) was defined/measured/monitored (logins, logfile analysis, etc.). Use/adoption metrics are important process outcomes that should be reported in any ehealth trial.

	1	2	3	4	5	
subitem not at all important	○	○	○	○	◉	essential

清除選取的項目

Does your paper address subitem 6a-ii?

Copy and paste relevant sections from manuscript text

The six-week follow-up evaluation data collections were completed via a web-based instrument after the invasive therapy. To prevent missing record, communication software (e.g., Line) was used to remind the participant's caregiver every week.
…
During six weeks follow up evaluation in experiment group(N=7), the average number of logins was 37.9 times (min.=14 times; max.=62 times) and the average number of played invasive therapies tasks was 58.2 times (min.=9; max.=179).

6a-iii) Describe whether, how, and when qualitative feedback from participants was obtained

Describe whether, how, and when qualitative feedback from participants was obtained (e.g., through emails, feedback forms, interviews, focus groups).

	1	2	3	4	5	
subitem not at all important	○	○	○	○	◉	essential

清除選取的項目

Does your paper address subitem 6a-iii?

Copy and paste relevant sections from manuscript text

In this study, the children therapeutic anxiety response was reported by the participants' family caregivers through web form which installed into their mobile phone when enrolled. …
"The fourth item: other response allowed family caregivers to free note any observation of the participants."

6b) Any changes to trial outcomes after the trial commenced, with reasons

Does your paper address CONSORT subitem 6b? *

Copy and paste relevant sections from the manuscript (include quotes in quotation marks "like this" to indicate direct quotes from your manuscript), or elaborate on this item by providing additional information not in the ms, or briefly explain why the item is not applicable/relevant for your study

No any changes to trial outcomes after the trial commenced

7a) How sample size was determined

NPT: When applicable, details of whether and how the clustering by care provides or centers was addressed

7a-i) Describe whether and how expected attrition was taken into account when calculating the sample size

Describe whether and how expected attrition was taken into account when calculating the sample size.

	1	2	3	4	5	
subitem not at all important	○	○	◉	○	○	essential

清除選取的項目

Does your paper address subitem 7a-i?

Copy and paste relevant sections from manuscript title (include quotes in quotation marks "like this" to indicate direct quotes from your manuscript), or elaborate on this item by providing additional information not in the ms, or briefly explain why the item is not applicable/relevant for your study

“In the study protocol drafting phase (e.g., since 2018), we adopted stratified random sampling and proposed samples size was thirty-four in each group according to the 2015-2016 Taiwan ALL incidence rate of each age and sex strata [3]”

7b) When applicable, explanation of any interim analyses and stopping guidelines

Does your paper address CONSORT subitem 7b? *

Copy and paste relevant sections from the manuscript (include quotes in quotation marks "like this" to indicate direct quotes from your manuscript), or elaborate on this item by providing additional information not in the ms, or briefly explain why the item is not applicable/relevant for your study

No any interim analyses and stopping guidelines

8a) Method used to generate the random allocation sequence
NPT: When applicable, how care providers were allocated to each trial group

Does your paper address CONSORT subitem 8a? *

Copy and paste relevant sections from the manuscript (include quotes in quotation marks "like this" to indicate direct quotes from your manuscript), or elaborate on this item by providing additional information not in the ms, or briefly explain why the item is not applicable/relevant for your study

“The eligible patients were screened by the researcher (DJ) before the recruitment in the study setting according to the 2015-2016 Taiwan ALL incidence rate of each age and sex strata[3]. Then each age and sex strata of participants was randomly assigned 1:1 into one of two study groups (control or experimental). The randomization scheme was generated by using the web site: www.randomization.com for each strata.”

8b) Type of randomisation; details of any restriction (such as blocking and block size)

Does your paper address CONSORT subitem 8b? *

Copy and paste relevant sections from the manuscript (include quotes in quotation marks "like this" to indicate direct quotes from your manuscript), or elaborate on this item by providing additional information not in the ms, or briefly explain why the item is not applicable/relevant for your study

“Participants were recruited by the researchers (DJ) and one trained research assistant in the pediatric hematology ward or outpatient clinic. The eligible patients were screened by the researcher (DJ) before the recruitment in the study setting according to the 2015-2016 Taiwan ALL incidence rate of each age and sex strata[3]. Then each age and sex strata of participants was randomly assigned 1:1 into one of two study groups (control or experimental). The randomization scheme was generated by using the web site: www.randomization.com for each strate.”

9) Mechanism used to implement the random allocation sequence (such as sequentially numbered containers), describing any steps taken to conceal the sequence until interventions were assigned

Does your paper address CONSORT subitem 9? *

Copy and paste relevant sections from the manuscript (include quotes in quotation marks "like this" to indicate direct quotes from your manuscript), or elaborate on this item by providing additional information not in the ms, or briefly explain why the item is not applicable/relevant for your study

“Participants were recruited by the researchers (DJ) and one trained research assistant in the pediatric hematology ward or outpatient clinic. The eligible patients were screened by the researcher (DJ) before the recruitment in the study setting according to the 2015-2016 Taiwan ALL incidence rate of each age and sex strata[3]. Then each age and sex strata of participants was randomly assigned 1:1 into one of two study groups (control or experimental). The randomization scheme was generated by using the web site: www.randomization.com for each strate.”

10) Who generated the random allocation sequence, who enrolled participants, and who assigned participants to interventions

Does your paper address CONSORT subitem 10? *

Copy and paste relevant sections from the manuscript (include quotes in quotation marks "like this" to indicate direct quotes from your manuscript), or elaborate on this item by providing additional information not in the ms, or briefly explain why the item is not applicable/relevant for your study

“Participants were recruited by the researchers (DJ) and one trained research assistant in the pediatric hematology ward or outpatient clinic. The eligible patients were screened by the researcher (DJ) before the recruitment in the study setting according to the 2015-2016 Taiwan ALL incidence rate of each age and sex strata[3]. Then each age and sex strata of participants was randomly assigned 1:1 into one of two study groups (control or experimental). The randomization scheme was generated by using the web site: www.randomization.com for each strate.”

11a) If done, who was blinded after assignment to interventions (for example, participants, care providers, those assessing outcomes) and how

NPT: Whether or not administering co-interventions were blinded to group assignment

11a-i) Specify who was blinded, and who wasn’t

Specify who was blinded, and who wasn’t. Usually, in web-based trials it is not possible to blind the participants [1, 3] (this should be clearly acknowledged), but it may be possible to blind outcome assessors, those doing data analysis or those administering co-interventions (if any).

	1	2	3	4	5	
subitem not at all important	○	○	○	○	◉	essential

清除選取的項目

Does your paper address subitem 11a-i? *

Copy and paste relevant sections from the manuscript (include quotes in quotation marks "like this" to indicate direct quotes from your manuscript), or elaborate on this item by providing additional information not in the ms, or briefly explain why the item is not applicable/relevant for your study

Patients were blinded whether they were in the experimental or control group.

11a-ii) Discuss e.g., whether participants knew which intervention was the "intervention of interest" and which one was the "comparator"

Informed consent procedures (4a-ii) can create biases and certain expectations - discuss e.g., whether participants knew which intervention was the "intervention of interest" and which one was the "comparator".

	1	2	3	4	5	
subitem not at all important	○	○	○	●	○	essential

清除選取的項目

Does your paper address subitem 11a-ii?

Copy and paste relevant sections from the manuscript (include quotes in quotation marks "like this" to indicate direct quotes from your manuscript), or elaborate on this item by providing additional information not in the ms, or briefly explain why the item is not applicable/relevant for your study

Patients were blinded whether they were in the experimental or control group.

11b) If relevant, description of the similarity of interventions

(this item is usually not relevant for ehealth trials as it refers to similarity of a placebo or sham intervention to a active medication/intervention)

Does your paper address CONSORT subitem 11b? *

Copy and paste relevant sections from the manuscript (include quotes in quotation marks "like this" to indicate direct quotes from your manuscript), or elaborate on this item by providing additional information not in the ms, or briefly explain why the item is not applicable/relevant for your study

No similarity of interventions in this study

12a) Statistical methods used to compare groups for primary and secondary outcomes

NPT: When applicable, details of whether and how the clustering by care providers or centers was addressed

Does your paper address CONSORT subitem 12a? *

Copy and paste relevant sections from the manuscript (include quotes in quotation marks "like this" to indicate direct quotes from your manuscript), or elaborate on this item by providing additional information not in the ms, or briefly explain why the item is not applicable/relevant for your study

“Frequency, percentage, mean, standard deviation was used for descriptive statistic for demographic data, and face rating scale. We compared the age, sex, risk classifications, treatment status, invasive therapies, face rating scale of the control and the experimental groups using Fisher's Exact test, Pearson's chi-square tests for categorical variables and Mann-Whitney U test for continuous variables. Significance was defined as P value < .05. For the other responses, each report was categorized as the positive and negative which defined by the research team with consensus. The positive response defined as the participants showed no cry or only a little nervous in the beginning of the therapy. The negative response defined as the participants showed cried, physical reaction (e.g., vomit) or any resistant behavior (e.g., try to run away).”

12a-i) Imputation techniques to deal with attrition / missing values

Imputation techniques to deal with attrition / missing values: Not all participants will use the intervention/comparator as intended and attrition is typically high in ehealth trials. Specify how participants who did not use the application or dropped out from the trial were treated in the statistical analysis (a complete case analysis is strongly discouraged, and simple imputation techniques such as LOCF may also be problematic [4]).

	1	2	3	4	5	
subitem not at all important	○	○	○	○	◉	essential

清除選取的項目

Does your paper address subitem 12a-i? *

Copy and paste relevant sections from the manuscript (include quotes in quotation marks "like this" to indicate direct quotes from your manuscript), or elaborate on this item by providing additional information not in the ms, or briefly explain why the item is not applicable/relevant for your study

All the primary outcome were self-reported and analyzed without missing values.

12b) Methods for additional analyses, such as subgroup analyses and adjusted analyses

Does your paper address CONSORT subitem 12b? *

Copy and paste relevant sections from the manuscript (include quotes in quotation marks "like this" to indicate direct quotes from your manuscript), or elaborate on this item by providing additional information not in the ms, or briefly explain why the item is not applicable/relevant for your study

No subgroup analyses and adjusted analyses

X26) REB/IRB Approval and Ethical Considerations [recommended as subheading under "Methods"] (not a CONSORT item)

X26-i) Comment on ethics committee approval

	1	2	3	4	5	
subitem not at all important	○	○	○	○	◉	essential

清除選取的項目

Does your paper address subitem X26-i?

Copy and paste relevant sections from the manuscript (include quotes in quotation marks "like this" to indicate direct quotes from your manuscript), or elaborate on this item by providing additional information not in the ms, or briefly explain why the item is not applicable/relevant for your study

“This study followed the ethical principles of the Declaration of Helsinki [18] and approved by the Institute of Review Board in National Taiwan University Hospital (IRB No. 201705014RINC) in Taiwan before trial started.”

x26-ii) Outline informed consent procedures

Outline informed consent procedures e.g., if consent was obtained offline or online (how? Checkbox, etc.?), and what information was provided (see 4a-ii). See [6] for some items to be included in informed consent documents.

	1	2	3	4	5	
subitem not at all important	○	○	○	○	◉	essential

清除選取的項目

Does your paper address subitem X26-ii?

Copy and paste relevant sections from the manuscript (include quotes in quotation marks "like this" to indicate direct quotes from your manuscript), or elaborate on this item by providing additional information not in the ms, or briefly explain why the item is not applicable/relevant for your study

“Participation in the study was voluntary. The eligible participants were provided two different informed consents according to their assigned groups. The consents included the same information on the participants’ cry scale and resist behavior data collection. Only the experiment groups’ consents provide the briefing information of the TVG.”

X26-iii) Safety and security procedures

Safety and security procedures, incl. privacy considerations, and any steps taken to reduce the likelihood or detection of harm (e.g., education and training, availability of a hotline)

	1	2	3	4	5	
subitem not at all important	○	○	○	◉	○	essential

清除選取的項目

Does your paper address subitem X26-iii?

Copy and paste relevant sections from the manuscript (include quotes in quotation marks "like this" to indicate direct quotes from your manuscript), or elaborate on this item by providing additional information not in the ms, or briefly explain why the item is not applicable/relevant for your study

To assess the reliability of TVG, three certified children art therapists were invited to preview the TVG. Then they completed one 12-item instrument which contain usefulness (9 items), trust (2 items) structured questions with five-point Likert scale (5: strongly agree; 1: strongly disagree) and narrative feedback (1 item) open question. The descriptive analysis of eleven structured items, content analysis of one narrative feedback item and internal consistence reliability (Cronbach α level) of three therapists were performed to evaluate the usefulness and trust of the TVG. The results were also used to optimize the TVG before phase 3.

RESULTS

13a) For each group, the numbers of participants who were randomly assigned, received intended treatment, and were analysed for the primary outcome

NPT: The number of care providers or centers performing the intervention in each group and the number of patients treated by each care provider in each center

Does your paper address CONSORT subitem 13a? *

Copy and paste relevant sections from the manuscript (include quotes in quotation marks "like this" to indicate direct quotes from your manuscript), or elaborate on this item by providing additional information not in the ms, or briefly explain why the item is not applicable/relevant for your study

Randomization and attrition data were organized according to CONSORT guideline [20] (see Figure 2). A total of 21 eligible ALL preschoolers, however, two of their family caregivers refused to participant the trial for concerning their child to use the electronic products. Two of the them was lost contact during the trial (e.g., miss their clinic visiting time). One family caregiver said her child (4 years and 10 months old) was not anxiety anymore when receiving the invasive therapy. The remaining of 16 eligible participants were enrolled and randomly allocated to the experimental (n = 8) or the control (n = 8) group. The recruitment rate was 76% (16/21). However, one of the participants in the experimental group was dropout for no participant's anxiety response report during six weeks follow-up evaluation. A total of 7 participants remained in the experimental group and 8 in the control group at the six weeks follow up."

13b) For each group, losses and exclusions after randomisation, together with reasons

Does your paper address CONSORT subitem 13b? (NOTE: Preferably, this is shown in a CONSORT flow diagram) *

Copy and paste relevant sections from the manuscript (include quotes in quotation marks "like this" to indicate direct quotes from your manuscript), or elaborate on this item by providing additional information not in the ms, or briefly explain why the item is not applicable/relevant for your study

"Randomization and attrition data were organized according to CONSORT guideline [20] (see Figure 2). A total of 21 eligible ALL preschoolers, however, two of their family caregivers refused to participant the trial for concerning their child to use the electronic products. Two of the them was lost contact during the trial (e.g., miss their clinic visiting time). One family caregiver said her child (4 years and 10 months old) was not anxiety anymore when receiving the invasive therapy. The remaining of 16 eligible participants were enrolled and randomly allocated to the experimental (n = 8) or the control (n = 8) group. The recruitment rate was 76% (16/21). However, one of the participants in the experimental group was dropout for no participant's anxiety response report during six weeks follow-up evaluation. A total of 7 participants remained in the experimental group and 8 in the control group at the six weeks follow up."

13b-i) Attrition diagram

Strongly recommended: An attrition diagram (e.g., proportion of participants still logging in or using the intervention/comparator in each group plotted over time, similar to a survival curve) or other figures or tables demonstrating usage/dose/engagement.

	1	2	3	4	5	
subitem not at all important	○	○	○	○	◉	essential

清除選取的項目

Does your paper address subitem 13b-i?

Copy and paste relevant sections from the manuscript or cite the figure number if applicable (include quotes in quotation marks "like this" to indicate direct quotes from your manuscript), or elaborate on this item by providing additional information not in the ms, or briefly explain why the item is not applicable/relevant for your study

“Randomization and attrition data were organized according to CONSORT guideline [20] (see Figure 2).”

14a) Dates defining the periods of recruitment and follow-up

Does your paper address CONSORT subitem 14a? *

Copy and paste relevant sections from the manuscript (include quotes in quotation marks "like this" to indicate direct quotes from your manuscript), or elaborate on this item by providing additional information not in the ms, or briefly explain why the item is not applicable/relevant for your study

“Data collection was from March, 2018 to April in 2020 by the study team.”

14a-i) Indicate if critical “secular events” fell into the study period

Indicate if critical “secular events” fell into the study period, e.g., significant changes in Internet resources available or “changes in computer hardware or Internet delivery resources”

	1	2	3	4	5	
subitem not at all important	○	○	○	○	◉	essential

清除選取的項目

Does your paper address subitem 14a-i?

Copy and paste relevant sections from the manuscript (include quotes in quotation marks "like this" to indicate direct quotes from your manuscript), or elaborate on this item by providing additional information not in the ms, or briefly explain why the item is not applicable/relevant for your study

No significant changes in Internet resources available

14b) Why the trial ended or was stopped (early)

Does your paper address CONSORT subitem 14b? *

Copy and paste relevant sections from the manuscript (include quotes in quotation marks "like this" to indicate direct quotes from your manuscript), or elaborate on this item by providing additional information not in the ms, or briefly explain why the item is not applicable/relevant for your study

Not Applicable - The trial did not end early.

15) A table showing baseline demographic and clinical characteristics for each group

NPT: When applicable, a description of care providers (case volume, qualification, expertise, etc.) and centers (volume) in each group

Does your paper address CONSORT subitem 15? *

Copy and paste relevant sections from the manuscript (include quotes in quotation marks "like this" to indicate direct quotes from your manuscript), or elaborate on this item by providing additional information not in the ms, or briefly explain why the item is not applicable/relevant for your study

The demographics from the two groups were similar (see Table 4). The largest age group was 5 years old (47%); sex group was boy (67%); risk classifications was standard (73%); treatment status was during their continuation stage (71%). There were no statistical differences which also homogeneity between experimental and control participants with regard to any of the demographic characteristics.

15-i) Report demographics associated with digital divide issues

In ehealth trials it is particularly important to report demographics associated with digital divide issues, such as age, education, gender, social-economic status, computer/Internet/ehealth literacy of the participants, if known.

	1	2	3	4	5	
subitem not at all important	○	○	○	○	◉	essential

清除選取的項目

Does your paper address subitem 15-i? *

Copy and paste relevant sections from the manuscript (include quotes in quotation marks "like this" to indicate direct quotes from your manuscript), or elaborate on this item by providing additional information not in the ms, or briefly explain why the item is not applicable/relevant for your study

“The demographics from the two groups were similar (see Table 4). The largest age group was 5 years old (47%); sex group was boy (67%); risk classifications was standard (73%); treatment status was during their continuation stage (71%). There were no statistical differences which also homogeneity between experimental and control participants with regard to any of the demographic characteristics.”

16) For each group, number of participants (denominator) included in each analysis and whether the analysis was by original assigned groups

16-i) Report multiple “denominators” and provide definitions

Report multiple “denominators” and provide definitions: Report N’s (and effect sizes) “across a range of study participation [and use] thresholds” [1], e.g., N exposed, N consented, N used more than x times, N used more than y weeks, N participants “used” the intervention/comparator at specific pre-defined time points of interest (in absolute and relative numbers per group). Always clearly define “use” of the intervention.

	1	2	3	4	5	
subitem not at all important	○	○	○	○	◉	essential

清除選取的項目

Does your paper address subitem 16-i? *

Copy and paste relevant sections from the manuscript (include quotes in quotation marks "like this" to indicate direct quotes from your manuscript), or elaborate on this item by providing additional information not in the ms, or briefly explain why the item is not applicable/relevant for your study

Table 7 showed that the mean FRS were 6.16 (SD= 3.08) among 67 records in experimental group, and 7.45 (SD = 2.71) among 69 records in control group. Mann-Whitney U test showed statistical difference (P = .036) between two groups (see Table 7). When receiving chemical medication administration therapy via IV, the mean FRS in experimental group was significantly lower than in control group (3.62 vs. 5.85, P = .038). The other five invasive therapies showed no difference between groups. The results of other responses showed that the experimental group had a little more positive responses and less negative responses than control group. (See Table 7)

16-ii) Primary analysis should be intent-to-treat

Primary analysis should be intent-to-treat, secondary analyses could include comparing only “users”, with the appropriate caveats that this is no longer a randomized sample (see 18-i).

	1	2	3	4	5	
subitem not at all important	○	○	○	○	◉	essential

清除選取的項目

Does your paper address subitem 16-ii?

Copy and paste relevant sections from the manuscript (include quotes in quotation marks "like this" to indicate direct quotes from your manuscript), or elaborate on this item by providing additional information not in the ms, or briefly explain why the item is not applicable/relevant for your study

Table 7 showed that the mean FRS were 6.16 (SD= 3.08) among 67 records in experimental group, and 7.45 (SD = 2.71) among 69 records in control group. Mann-Whitney U test showed statistical difference (P = .036) between two groups (see Table 7). When receiving chemical medication administration therapy via IV, the mean FRS in experimental group was significantly lower than in control group (3.62 vs. 5.85, P = .038). The other five invasive therapies showed no difference between groups. The results of other responses showed that the experimental group had a little more positive responses and less negative responses than control group. (See Table 7)

17a) For each primary and secondary outcome, results for each group, and the estimated effect size and its precision (such as 95% confidence interval)

Does your paper address CONSORT subitem 17a? *

Copy and paste relevant sections from the manuscript (include quotes in quotation marks "like this" to indicate direct quotes from your manuscript), or elaborate on this item by providing additional information not in the ms, or briefly explain why the item is not applicable/relevant for your study

The statistical power of 15 participants was 0.23 which was not adequate according to Cohen [25] and might affect the credibility of the results of this study.

17a-i) Presentation of process outcomes such as metrics of use and intensity of use

In addition to primary/secondary (clinical) outcomes, the presentation of process outcomes such as metrics of use and intensity of use (dose, exposure) and their operational definitions is critical. This does not only refer to metrics of attrition (13-b) (often a binary variable), but also to more continuous exposure metrics such as “average session length”. These must be accompanied by a technical description how a metric like a “session” is defined (e.g., timeout after idle time) [1] (report under item 6a).

	1	2	3	4	5	
subitem not at all important	○	○	○	○	◉	essential

清除選取的項目

Does your paper address subitem 17a-i?

Copy and paste relevant sections from the manuscript (include quotes in quotation marks "like this" to indicate direct quotes from your manuscript), or elaborate on this item by providing additional information not in the ms, or briefly explain why the item is not applicable/relevant for your study

According to the participants’ FRS reports, the internal consistence reliability of FRS was 0.52 (e.g., Cronbach’s Alpha) which indicated the acceptable reliability [19]. Table 7 showed that the mean FRS were 6.16 (SD= 3.08) among 67 records in experimental group, and 7.45 (SD = 2.71) among 69 records in control group. Mann-Whitney U test showed statistical difference (P = .036) between two groups (see Table 7). When receiving chemical medication administration therapy via IV, the mean FRS in experimental group was significantly lower than in control group (3.62 vs. 5.85, P = .038). The other five invasive therapies showed no difference between groups. The results of other responses showed that the experimental group had a little more positive responses and less negative responses than control group. (See Table 7)

17b) For binary outcomes, presentation of both absolute and relative effect sizes is recommended

Does your paper address CONSORT subitem 17b? *

Copy and paste relevant sections from the manuscript (include quotes in quotation marks "like this" to indicate direct quotes from your manuscript), or elaborate on this item by providing additional information not in the ms, or briefly explain why the item is not applicable/relevant for your study

Not applicable on binary outcomes presentation of both absolute and relative effect sizes

18) Results of any other analyses performed, including subgroup analyses and adjusted analyses, distinguishing pre-specified from exploratory

Does your paper address CONSORT subitem 18? *

Copy and paste relevant sections from the manuscript (include quotes in quotation marks "like this" to indicate direct quotes from your manuscript), or elaborate on this item by providing additional information not in the ms, or briefly explain why the item is not applicable/relevant for your study

Not applicable on any other analyses performed, including subgroup analyses and adjusted analyses, distinguishing pre-specified from exploratory

18-i) Subgroup analysis of comparing only users

A subgroup analysis of comparing only users is not uncommon in ehealth trials, but if done, it must be stressed that this is a self-selected sample and no longer an unbiased sample from a randomized trial (see 16-iii).

	1	2	3	4	5	
subitem not at all important	(●)	( )	( )	( )	( )	essential

清除選取的項目

Does your paper address subitem 18-i?

Copy and paste relevant sections from the manuscript (include quotes in quotation marks "like this" to indicate direct quotes from your manuscript), or elaborate on this item by providing additional information not in the ms, or briefly explain why the item is not applicable/relevant for your study

Not applicable on subgroup analysis of comparing only users

19) All important harms or unintended effects in each group
(for specific guidance see CONSORT for harms)

Does your paper address CONSORT subitem 19? *

Copy and paste relevant sections from the manuscript (include quotes in quotation marks "like this" to indicate direct quotes from your manuscript), or elaborate on this item by providing additional information not in the ms, or briefly explain why the item is not applicable/relevant for your study

“Although the TVG could decrease the therapeutic anxiety for ALL children, there still existing the potential negative concern by their family caregivers. A total of three family caregivers from the eligible participants rejected this trial, most (e.g., 2/3) of them avoid their child to use the video games.

19-i) Include privacy breaches, technical problems

Include privacy breaches, technical problems. This does not only include physical “harm” to participants, but also incidents such as perceived or real privacy breaches [1], technical problems, and other unexpected/unintended incidents. “Unintended effects” also includes unintended positive effects [2].

	1	2	3	4	5	
subitem not at all important	◉	○	○	○	○	essential

清除選取的項目

Does your paper address subitem 19-i?

Copy and paste relevant sections from the manuscript (include quotes in quotation marks "like this" to indicate direct quotes from your manuscript), or elaborate on this item by providing additional information not in the ms, or briefly explain why the item is not applicable/relevant for your study

No privacy breaches, technical problems

19-ii) Include qualitative feedback from participants or observations from staff/researchers

Include qualitative feedback from participants or observations from staff/researchers, if available, on strengths and shortcomings of the application, especially if they point to unintended/unexpected effects or uses. This includes (if available) reasons for why people did or did not use the application as intended by the developers.

	1	2	3	4	5	
subitem not at all important	○	○	○	○	◉	essential

清除選取的項目

Does your paper address subitem 19-ii?

Copy and paste relevant sections from the manuscript (include quotes in quotation marks "like this" to indicate direct quotes from your manuscript), or elaborate on this item by providing additional information not in the ms, or briefly explain why the item is not applicable/relevant for your study

The results of other responses showed that the experimental group had a little more positive responses and less negative responses than control group.

DISCUSSION

22) Interpretation consistent with results, balancing benefits and harms, and considering other relevant evidence

NPT: In addition, take into account the choice of the comparator, lack of or partial blinding, and unequal expertise of care providers or centers in each group

22-i) Restate study questions and summarize the answers suggested by the data, starting with primary outcomes and process outcomes (use)

Restate study questions and summarize the answers suggested by the data, starting with primary outcomes and process outcomes (use).

	1	2	3	4	5	
subitem not at all important	○	○	○	○	◉	essential

清除選取的項目

Does your paper address subitem 22-i? *

Copy and paste relevant sections from the manuscript (include quotes in quotation marks "like this" to indicate direct quotes from your manuscript), or elaborate on this item by providing additional information not in the ms, or briefly explain why the item is not applicable/relevant for your study

During six weeks follow-up evaluation, the average number of logins (37.5 times), played tasks (76 times) in the experimental group showed that the acceptance and reliability of using TVG and not related to their treatment status (ρ=-.138, P=.699). The results indicated that the participants' caregiver (e.g., parents) may trust TVG and had the willingness to allow their sick child to use TVG. Meanwhile, TVG followed the protocol TOPG-ALL 2013 and could facilitate any status of their treatment.
With caregiver-report children anxiety scale, the principal results showed the experimental group had significant improvement in their FRS (6.16 vs 7.45, P= .036) and less negative response (4 vs.13) compared to the control group. It was indicated that our TVG had benefit on reduction of therapeutic anxiety.

22-ii) Highlight unanswered new questions, suggest future research

Highlight unanswered new questions, suggest future research.

	1	2	3	4	5	
subitem not at all important	○	○	○	○	◉	essential

清除選取的項目

Does your paper address subitem 22-ii?

Copy and paste relevant sections from the manuscript (include quotes in quotation marks "like this" to indicate direct quotes from your manuscript), or elaborate on this item by providing additional information not in the ms, or briefly explain why the item is not applicable/relevant for your study

“The effective results of the current data, it was recommended to include more cancer related education in TVG such as medication taking, hand hygrine, tooth washing to facilitate preschooler with ALL. In addition, the TVG could also introduced to the other common cancers (e.g., brain tumor, osteosarcoma) of children to help them to release their anxiety during difficult treatment. The health education knowledge that could facilitate the family member to take care of their sick child or siblings could also be provided when design the next TVG.”

20) Trial limitations, addressing sources of potential bias, imprecision, and, if relevant, multiplicity of analyses

20-i) Typical limitations in ehealth trials

Typical limitations in ehealth trials: Participants in ehealth trials are rarely blinded. Ehealth trials often look at a multiplicity of outcomes, increasing risk for a Type I error. Discuss biases due to non-use of the intervention/usability issues, biases through informed consent procedures, unexpected events.

	1	2	3	4	5	
subitem not at all important	○	○	○	○	◉	essential

清除選取的項目

Does your paper address subitem 20-i? *

Copy and paste relevant sections from the manuscript (include quotes in quotation marks "like this" to indicate direct quotes from your manuscript), or elaborate on this item by providing additional information not in the ms, or briefly explain why the item is not applicable/relevant for your study

In the study protocol drafting phase (e.g., since 2018), we adopted stratified random sampling and proposed samples size was thirty-four in each group according to the 2015-2016 Taiwan ALL incidence rate of each age and sex strata [3]. However, in Taiwan, the total number of births (165 thousand) was first become lower than the total number of deaths (173 thousand) since 2020 [24]. Therefore, it may cause the lower incidence rate of ALL preschoolers during this trial (2018-2020). The statistical power of 15 participants was 0.23 which was not adequate according to Cohen [25] and might affect the credibility of the results of this study.
There might be the limitation regarded to the participant blindness procedure in this study. The primary caregivers of these participants knew their assigned group according to our informed consent process. To our knowledge, the effects on the non-blindness preschoolers' caregivers cause the self-report bias in the clinical trial study were not clear. Therefore, the therapeutical anxiety improvement after using our TVG was conservatively inferred.

21) Generalisability (external validity, applicability) of the trial findings

NPT: External validity of the trial findings according to the intervention, comparators, patients, and care providers or centers involved in the trial

21-i) Generalizability to other populations

Generalizability to other populations: In particular, discuss generalizability to a general Internet population, outside of a RCT setting, and general patient population, including applicability of the study results for other organizations

	1	2	3	4	5	
subitem not at all important	○	○	○	○	●	essential

清除選取的項目

Does your paper address subitem 21-i?

Copy and paste relevant sections from the manuscript (include quotes in quotation marks "like this" to indicate direct quotes from your manuscript), or elaborate on this item by providing additional information not in the ms, or briefly explain why the item is not applicable/relevant for your study

The purpose of the study was to investigate the therapeutic anxiety response for preschoolers with ALL in Taiwan after using innovative TVG designed by our team. According to the results, TVG users decreased their anxiety after receiving cancer invasive therapies than non-users. It might because the TVG deliver the simulation invasive procedures through character role rehearsal. When the subjects perceived the positive feedback from these tasks, the negative perception (e.g., therapeutic anxiety) perhaps be cognitive/attentional distracted.

21-ii) Discuss if there were elements in the RCT that would be different in a routine application setting

Discuss if there were elements in the RCT that would be different in a routine application setting (e.g., prompts/reminders, more human involvement, training sessions or other co-interventions) and what impact the omission of these elements could have on use, adoption, or outcomes if the intervention is applied outside of a RCT setting.

	1	2	3	4	5	
subitem not at all important	○	○	○	○	◉	essential

清除選取的項目

Does your paper address subitem 21-ii?

Copy and paste relevant sections from the manuscript (include quotes in quotation marks "like this" to indicate direct quotes from your manuscript), or elaborate on this item by providing additional information not in the ms, or briefly explain why the item is not applicable/relevant for your study

According to the pervious systematic review study, the therapeutic anxiety improvement (see Table 7) in our experimental group was consistent with the results of play therapy reducing anxiety during the hospital stay in different countries [21]. It was also similar to the study which reducing of preoperative anxiety for children with 5-11 ages in Jordan [22]. This may be due to the TVG was designed for the pre-operations stage of cognitive development of the preschoolers according to the Piaget which indicated that the participants could use the TVG easily without logical thinking [17]. The content of TVG also provide the simulation according to the real invasive therapies during the ALL treatments and increasing the participants' familiarity by playing the TVG repeatedly.
Although the TVG could decrease the therapeutic anxiety for ALL children, there still existing the potential negative concern by their family caregivers. A total of three family caregivers from the eligible participants rejected this trial, most (e.g., 2/3) of them avoid their child to use the video games. To our knowledge, there was increasing amount of literature that focused on pathological and non-pathological correlates of video game playing, with specific attention towards internet gaming disorder (IGD) [23]. It may cause of the trustless to the TVG for these family caregivers.

OTHER INFORMATION

23) Registration number and name of trial registry

Does your paper address CONSORT subitem 23? *

Copy and paste relevant sections from the manuscript (include quotes in quotation marks "like this" to indicate direct quotes from your manuscript), or elaborate on this item by providing additional information not in the ms, or briefly explain why the item is not applicable/relevant for your study

Trial Registration: NCT04199637

24) Where the full trial protocol can be accessed, if available

Does your paper address CONSORT subitem 24? *

Cite a Multimedia Appendix, other reference, or copy and paste relevant sections from the manuscript (include quotes in quotation marks "like this" to indicate direct quotes from your manuscript), or elaborate on this item by providing additional information not in the ms, or briefly explain why the item is not applicable/relevant for your study

Trial Registration: NCT04199637

25) Sources of funding and other support (such as supply of drugs), role of funders

Does your paper address CONSORT subitem 25? *

Copy and paste relevant sections from the manuscript (include quotes in quotation marks "like this" to indicate direct quotes from your manuscript), or elaborate on this item by providing additional information not in the ms, or briefly explain why the item is not applicable/relevant for your study

We appreciate the funding from the Ministry of Science and Technology (MOST 108-2314-B-010-03)

X27) Conflicts of Interest (not a CONSORT item)

X27-i) State the relation of the study team towards the system being evaluated

In addition to the usual declaration of interests (financial or otherwise), also state the relation of the study team towards the system being evaluated, i.e., state if the authors/evaluators are distinct from or identical with the developers/sponsors of the intervention.

	1	2	3	4	5	
subitem not at all important	○	○	○	○	◉	essential

清除選取的項目

Does your paper address subitem X27-i?

Copy and paste relevant sections from the manuscript (include quotes in quotation marks "like this" to indicate direct quotes from your manuscript), or elaborate on this item by providing additional information not in the ms, or briefly explain why the item is not applicable/relevant for your study

Conflicts of Interest
None declared.

About the CONSORT EHEALTH checklist

As a result of using this checklist, did you make changes in your manuscript? *

- ○ yes, major changes
- ○ yes, minor changes
- ◉ no

What were the most important changes you made as a result of using this checklist?

您的回答

How much time did you spend on going through the checklist INCLUDING making changes in your manuscript *

2 hours on going through the checklist

As a result of using this checklist, do you think your manuscript has improved? *

- ○ yes
- ◉ no
- ○ 其他：

Would you like to become involved in the CONSORT EHEALTH group?

This would involve for example becoming involved in participating in a workshop and writing an "Explanation and Elaboration" document

- ◉ yes
- ○ no
- ○ 其他：

清除選取的項目

Any other comments or questions on CONSORT EHEALTH

您的回答

STOP - Save this form as PDF before you click submit

To generate a record that you filled in this form, we recommend to generate a PDF of this page (on a Mac, simply select "print" and then select "print as PDF") before you submit it.

When you submit your (revised) paper to JMIR, please upload the PDF as supplementary file.

Don't worry if some text in the textboxes is cut off, as we still have the complete information in our database. Thank you!

Final step: Click submit !

Click submit so we have your answers in our database!

提交

清除表單

請勿利用 Google 表單送出密碼。

Google 並未認可或建立這項內容。 檢舉濫用情形 - 服務條款 - 隱私權政策

Google 表單
