# Supplementary material for: Correction: Development of a Therapeutic Video Game With the MDA Framework to Decrease Anxiety in Preschool-Aged Children With Acute Lymphoblastic Leukemia: Mixed Methods Approach
Source: JMIR Serious Games. 2022 Oct 5;10(4):e43211. doi: 10.2196/43211 (PMC9582910; doi:10.2196/43211)
Supplement: Multimedia Appendix 1 [file games_v10i4e43211_app1.docx]

**Table 3.** Caregiver-reported invasive therapies.

| Invasive therapy administered | Experimental group (n=7) | | | | Control group (n=8) | | | *P* value^a^ |
| --- | --- | --- | --- | --- | --- | --- | --- | --- |
|  | Times administered, n (%) | FRS^b^ score, range | FRS score, mean (SD) | Times administered, n (%) | | FRS score, range | FRS score, mean (SD) |  |
|  | | | | | | | | |
| IM^c^ injection (buttocks injection) | 25 (37) | 1-5 | 3.5 (1.6) | 27 (39) | | 0-6 | 3.9 (1.3) | .81 |
| PORT^d^ puncture | 17 (25) | 0-6 | 2.8 (1.9) | 18 (26) | | 1-4 | 2.3 (1.0) | .90 |
| IV^e^ injection | 13 (19) | 1-6 | 1.9 (1.9) | 13 (19) | | 0-3 | 2.1 (0.4) | .50 |
| IT^f^ injection | 6 (9) | 0-2 | 2 (0) | 8 (12) | | 0-2 | 1.1 (0.4) | .66 |
| BMA^g^ | 4 (6) | 0-2 | 2 (0) | 3 (4) | | 0-2 | 1.5 (0.7) | .77 |
| BT^h^ | 2 (3) | 0-2 | 2 (0) | 0 (0) | | 0-0 | 0 (0) | .29 |
| Total | 67 (100) | 6-15 | 9.6 (3.5) | 69 (100) | | 2-11 | 8.6 (2.9) | >.99 |

^a^This *P* value was based on the Mann-Whitney *U* test.

^b^FRS: face rating scale.

^c^IM: intramuscular.

^d^PORT: port-a-cath catheter system.

^e^IV: intravenous.

^f^IT: intrathecal.

^g^BMA: bone marrow aspiration.

^h^BT: blood transfusion.
